# The Microbiota Mediates Pathogen Clearance from the Gut Lumen after Non-Typhoidal *Salmonella* Diarrhea

**DOI:** 10.1371/journal.ppat.1001097

**Published:** 2010-09-09

**Authors:** Kathrin Endt, Bärbel Stecher, Samuel Chaffron, Emma Slack, Nicolas Tchitchek, Arndt Benecke, Laurye Van Maele, Jean-Claude Sirard, Andreas J. Mueller, Mathias Heikenwalder, Andrew J. Macpherson, Richard Strugnell, Christian von Mering, Wolf-Dietrich Hardt

**Affiliations:** 1 Institute of Microbiology, ETH Zürich, Zürich, Switzerland; 2 Institute of Molecular Biology and Swiss Institute of Bioinformatics, University of Zürich, Zürich, Switzerland; 3 Gastroenterology Inselspital, Department Klinische Forschung, Bern, Switzerland; 4 Institut des Hautes Études Scientifiques & CNRS USR3078, Bures sur Yvette, France; 5 Institut National de la Santé et de la Recherche Médicale, U801; Institut Pasteur de Lille; Univ. Lille Nord de France, UDSL, Lille, France; 6 Institute of Neuropathology, University Hospital of Zurich, Zürich, Switzerland; 7 Department of Microbiology and Immunology, The University of Melbourne, Parkville, Victoria, Australia; The Rockefeller University, United States of America

## Abstract

Many enteropathogenic bacteria target the mammalian gut. The mechanisms protecting the host from infection are poorly understood. We have studied the protective functions of secretory antibodies (sIgA) and the microbiota, using a mouse model for *S. typhimurium* diarrhea. This pathogen is a common cause of diarrhea in humans world-wide. *S. typhimurium* (*S. tm* ^att^, *sseD*) causes a self-limiting gut infection in streptomycin-treated mice. After 40 days, all animals had overcome the disease, developed a sIgA response, and most had cleared the pathogen from the gut lumen. sIgA limited pathogen access to the mucosal surface and protected from gut inflammation in challenge infections. This protection was O-antigen specific, as demonstrated with pathogens lacking the *S. typhimurium* O-antigen (*wbaP*, *S. enteritidis*) and sIgA-deficient mice (TCRβ^−/−^δ^−/−^, J_H_^−/−^, IgA^−/−^, pIgR^−/−^). Surprisingly, sIgA-deficiency did not affect the kinetics of pathogen clearance from the gut lumen. Instead, this was mediated by the microbiota. This was confirmed using ‘L-mice’ which harbor a low complexity gut flora, lack colonization resistance and develop a normal sIgA response, but fail to clear *S. tm* ^att^ from the gut lumen. In these mice, pathogen clearance was achieved by transferring a normal complex microbiota. Thus, besides colonization resistance ( = pathogen blockage by an intact microbiota), the microbiota mediates a second, novel protective function, i.e. pathogen clearance. Here, the normal microbiota re-grows from a state of depletion and disturbed composition and gradually clears even very high pathogen loads from the gut lumen, a site inaccessible to most “classical” immune effector mechanisms. In conclusion, sIgA and microbiota serve complementary protective functions. The microbiota confers colonization resistance and mediates pathogen clearance in primary infections, while sIgA protects from disease if the host re-encounters the same pathogen. This has implications for curing *S. typhimurium* diarrhea and for preventing transmission.

## Author Summary

Numerous pathogens infect the gut. Protection against these infections is mediated by mucosal immune defenses including secreted IgA as well as by the competing intestinal microbiota. However, so far the relative importance of these two different defense mechanisms remains unclear. We addressed this question using the example of non-typhoidal *Salmonella* (NTS) gut infections which can be spread in stool of infected patients over long periods of time. We used a mouse model to reveal that the intestinal microbiota and the adaptive immune system hold different but complementary functions in fighting NTS infections. A primary *Salmonella* infection disrupts the normal microbiota and elicits *Salmonella*-specific sIgA. sIgA prevents disease when the animal is infected with NTS for a second time. However, sIgA was dispensable for pathogen clearance from the gut. Instead, this was mediated by the microbiota. By re-establishing its normal density and composition, the microbiota was necessary and sufficient for terminating long-term fecal *Salmonella* excretion. This establishes a novel paradigm: The microbiota clears the pathogen from the gut lumen, while sIgA protects from disease upon re-infection with the same pathogen. This has implications for the evolutionary role of sIgA responses as well as for developing microbiota-based therapies for curing infected patients.

## Introduction

Bacterial diarrhea is a global cause of morbidity and mortality. In most cases, the acute disease symptoms cease after a few days and the pathogen is eliminated from the gut. However, the mechanisms eliminating enteropathogenic bacteria from the gut lumen are poorly understood. Most “classical” effector mechanisms of the immune system are ineffective in the gut lumen (i.e. complement-mediated killing, opsonophagocytosis, T-cell mediated toxicity). In the gut, innate and adaptive immune responses such as antimicrobial peptides, natural and pathogen-specific mucosal secretory IgA (sIgA) antibodies are considered to be cardinal defense mechanisms. In addition to the host's immune system, the highly dense and diverse bacterial community in the gut (the microbiota; >500 different species [1], [2]) plays a key role by inhibiting pathogen growth in the gut lumen right from the beginning. This phenomenon is referred to as ‘colonization resistance’ and efficiently blocks infections by *Clostridium difficile*, *Salmonella* spp. and many other pathogenic bacteria [3]. Colonization resistance might be based on nutrient limitation, release of inhibitory metabolites, production of bactericidal compounds, the competition for binding sites and other, unidentified features of the dense microbial community [4], [5].

Much less is known about the mechanisms clearing enteropathogenic bacteria from the gut lumen once they have established an infection in this niche. ‘Pathogen clearance’ differs significantly from colonization resistance as both, the mucosa [6] and the microbiota, must recover from pathogen-inflicted disturbance while eliminating the pathogen [7]. Here, we have studied the mechanisms of pathogen clearance from the gut lumen using the example of non-typhoidal *Salmonella* (NTS) diarrhea.

NTS infections, including *S. enterica* spp. I serovar Typhimurium (*S. tm*), account for a significant share of food-borne diarrhea in Europe and Northern America. In sub-Saharan Africa, NTS are also an important cause of invasive disease with high mortality, particularly in HIV infected individuals [8]. In humans, colonization resistance confers partial protection, but antibiotic treatment increases the risk of *Salmonella* diarrhea [9], [10]. In the typical cases of NTS diarrhea, the pathogen begins to grow in the gut and disease symptoms manifest eight to 24h after consumption of contaminated food or water. Usually, the pathogen remains limited to the gastrointestinal tract and diarrhea subsides within several days. After cessation of symptoms, *Salmonella* remains detectable in the stool for weeks, several months or sometimes even longer [11], [12]. Pathogen clearance seems to fail in these long-term ‘asymptomatic excretors’. This is problematic, as ‘asymptomatic excretors’ pose a significant risk of transmission, in particular when food workers in restaurants or the food industry are affected [13].

So far, we can only speculate about mechanisms mediating pathogen clearance from the gut lumen. Antimicrobial peptides might be involved in some infections, but should not affect *S. tm* clearance, as this pathogen is particularly resistant against this type of compound [14], [15]. Antibody responses, i.e. pathogen-specific secretory IgA (sIgA), might also clear pathogens from the gut lumen. *S. tm* elicits profound antibody responses against LPS and protein antigens [16]. In systemic infection models antibody responses can confer some degree of protection [17], [18]. Previous work on the role of sIgA in intestinal *S. tm* infection yielded conflicting results. sIgA protected cultured epithelial cells from *S. tm* infection, but did not reduce intestinal pathogen densities [18]. Similar findings were made for the enteropathogenic bacterium *Citrobacter rodentium* [19]. However, the role of sIgA in pathogen clearance in models of acute *Salmonella* enterocolitis with high intestinal pathogen loads has not been addressed so far. Finally, we reasoned that the microbiota itself might contribute to pathogen clearance. It remained to be shown which mechanisms contribute to pathogen clearance.

We have used a *Salmonella* diarrhea mouse model to analyze the relative importance of sIgA and the intestinal microbiota in *S. tm* clearance after infection. In mice, the intestinal microbiota confers colonization resistance. Normally, <10% of mice permit pathogen growth and get mucosal inflammation upon oral *S. tm* infection [20]. Oral antibiotic-treatment alleviates colonization resistance and wild type *S. tm* grows up to very high densities in the intestinal lumen and induces mucosal inflammation (colitis) in 100% of the animals [6]. The gut inflammation allows *S. tm* to out-compete the microbiota thus promoting pathogen overgrowth [21]. Here, we have extended this mouse model to study pathogen clearance at later phases of the primary infection when acute mucosal inflammation has ceased. We analyzed the levels of pathogen shedding, sIgA responses and the role of the microbiota. This revealed that the microbiota plays an essential role in pathogen clearance. The implications for curing asymptomatic excretors and preventing *S. tm* diarrhea are discussed.

## Results

### Sm-treated mice recover from *S. tm* ^att^ induced gut inflammation and display extensive differences in the kinetics of fecal pathogen clearance

In sm-treated mice, infection with an attenuated *S. typhimurium* strain (*S. typhimurium* SL1344 *sseD*; termed *S. tm* ^att^; Table S1) is known to recapitulate key aspects of the early stages of human NTS diarrhea, i.e. gut inflammation 8h after orogastric exposure with infection confined to the gastrointestinal tract [22]. Symptoms of the acute gut inflammation usually decline by 5–7 days after infection [23]. In order to assess, if this model may be useful to dissect the role of pathogen-specific sIgA and the intestinal microbiota in pathogen clearance at the final stage of a primary infection we analyzed the outcome of long-term *S. tm* ^att^ infections [6], [24].

**Figure 1:**
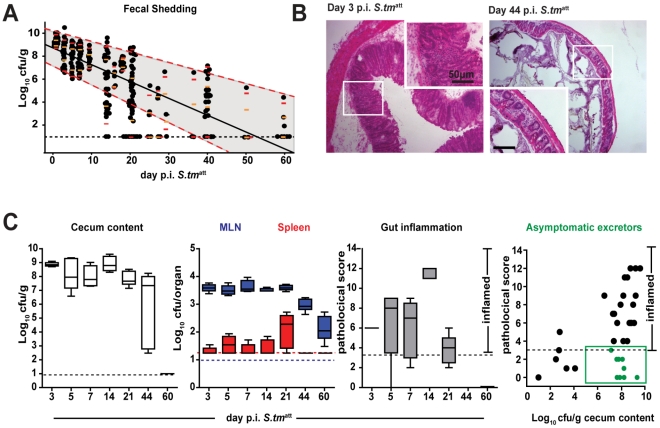
*S. tm* ^att^ infection yields ‘asymptomatic excretors’ and elicits O-antigen specific immunity. **A**. Time course of fecal *S. tm* ^att^ shedding. Sm-treated mice were infected with *S. tm* ^att^ and fecal *Salmonella* shedding was monitored (5<n<48 individual mice per time point). Medians are shown in black; orange and red: 5%, 25%, 75% and 95% quantiles. Dashed red lines: weighted linear regression on the 5% and 95% quantiles. **B**. H&E stained cross-section of the cecum at day 3 (left) and day 44 (right) post *S. tm* ^att^ infection. Enlarged section (white box) is shown in inset. Scale bar: 50µm. **C**. *S. tm* ^att^ loads in cecum (left panel; Log_10_ cfu/g), spleen (red) and MLN (blue; both Log_10_ cfu/organ; 2^nd^ panel) and cecal mucosa inflammation (3^rd^ panel) at the indicated times post *S. tm* ^att^ infection. ‘Asymptomatic excretors’ (4^th^ panel) are defined as showing a pathological score ≤3 while shedding ≥10^5^ cfu/g *S. tm* ^att^.(green box). Each dot represents an individual mouse between day 3 and 60 *S. tm* ^att^ immunization. Black dotted line: detection limit.

We monitored *S. tm* ^att^ shedding for up to 60 days after infection. *S. tm* ^att^ shedding in stool began to decrease after a few days, varied extensively between different animals and lasted for 2 to 8 weeks (Fig. 1A). At 60 day p.i., *S. tm* ^att^ shedding was reduced below 10^5^ cfu/g (p<0.05 day 1 vs. day 60 p.i.). At all stages, the infection remained largely confined to the gastrointestinal tract and draining mesenteric lymph nodes (MLN) and gut inflammation subsided after 7–44 days (Fig. 1B,C). Interestingly, we observed a high incidence of ‘asymptomatic excretors’ around day 44 post infection (p.i.). These mice were characterized by a low pathological score (≤3) and high cecum pathogen loads (≥10^5^ cfu/g stool; Fig. 1C, right panel, green symbols). This may indicate that pathogen clearance from the gut lumen is not necessary in order to resolve gut inflammation. In fact, both might be independent from each other. We concluded that this model could be useful to analyze the mechanism of pathogen clearance from the gut lumen after infection.

### *S. tm* ^att^ induces O-antigen specific mucosal protection

**Figure 2:**
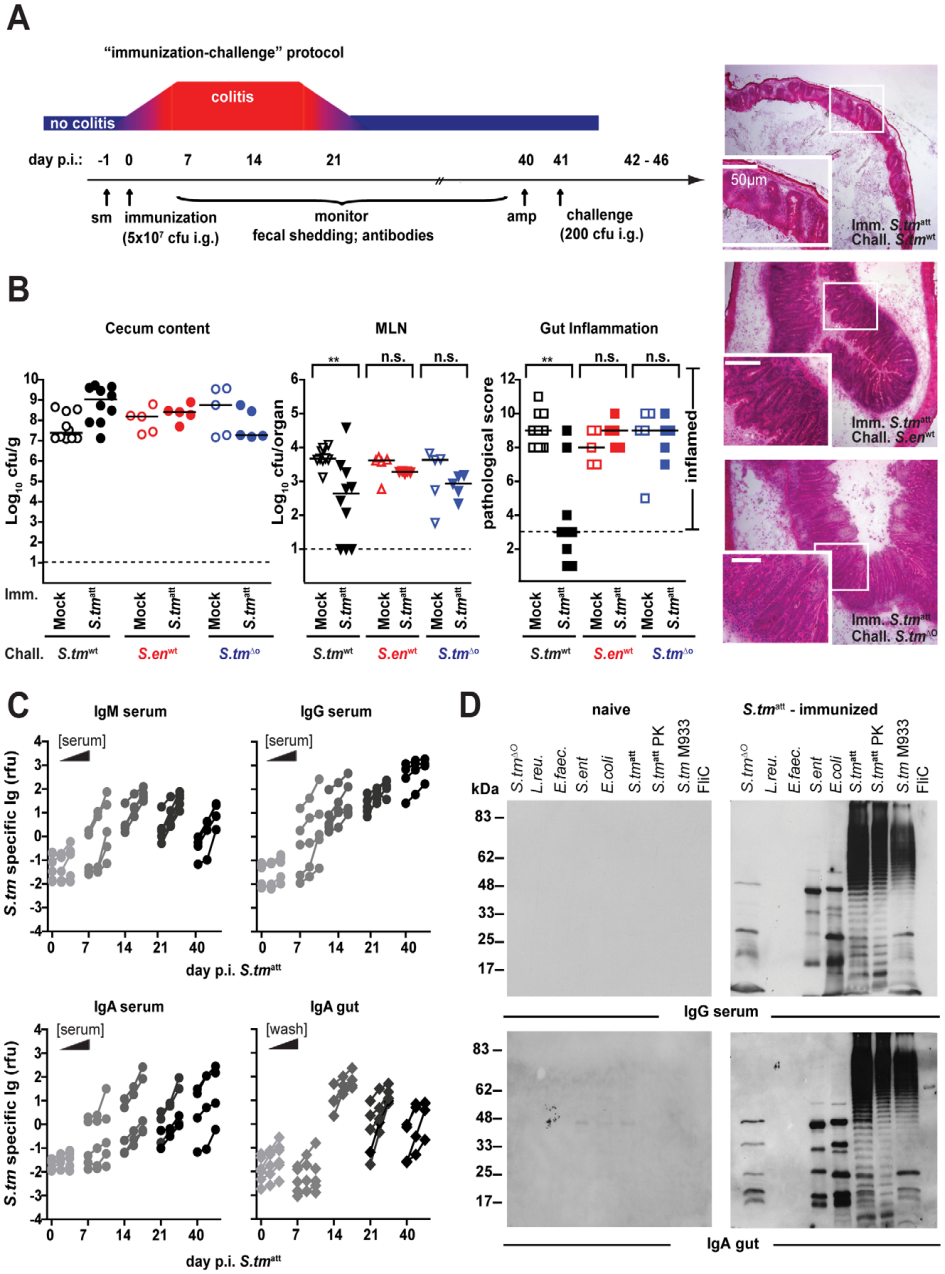
*S. tm* ^att^ induces O-antigen specific mucosal protection. **A**. ‘Immunization-challenge’ model. At day 0, sm-treated mice are infected and *S. tm* ^att^ (5×10^7^cfu; i.g.). They develop enteric pathology (red) that usually declines by day 14–20 p.i. (blue). Afterwards, the mice appear healthy. At day 40 p.i., mice are treated with ampicillin (20mg i.g.) and challenged with *S. tm* ^wt^ (or other strains; 200cfu i.g.). The degree of *Salmonella*-induced gut inflammation and pathogen loads are determined at day 1–5 post challenge. **B**. *S. tm* ^att^ immunized mice are protected in an O-antigen dependent fashion. *S. tm* ^att^ (filled symbols) or mock (open symbols) immunized mice were challenged with *S. tm* ^wt^ (black; n = 10), *S. en* ^wt^ (red circles; n = 5) or *S. tm* ^ΔO^ (blue; n = 5) at day 40 post immunization. At day 2 post challenge, challenge strain loads were assessed in the cecum content (left panel) and the MLN (middle panel). Dashed lines indicate detection limits. Right panels: inflammation of the cecal mucosa (score≤3 indicates no inflammation). Black bar: median; *p<0.05; **p<0.005; n.s. = not significant. H&E stained cross-sections of the cecum of *S. tm* ^att^-immunized mice at day 2 post challenge with *S. tm* ^wt^ (upper panel), *S. en* ^wt^ (middle panel) and *S. tm* ^ΔO^ (lower panel). Enlarged section (white box) is shown in the lower panel. Scale bar: 50µm. **C**. Time course of the *S. tm* ^att^ specific humoral immune response. Antibodies directed against the surface of *S. tm* ^att^ in serum or gut wash of the *S. tm* ^att^ -immunized mice from Fig. 1C were analyzed by bacterial FACS (Materials and Methods; Fig.S1). The Y-axis shows *S. tm* ^att^ specifc Ig (relative fluorescence units; rfu), the X-axis different dilutions of serum or gut wash (1∶ 20, 1∶60, 1∶180) at different days post *S. tm^att^* immunization. **D**. *S. tm* ^att^-immunized mice mount an O-antigen specific antibody response. Serum and gut wash Ig from naïve and *S. tm* ^att^ infected mice (day 40 post infection) were analyzed by immunoblot against different bacterial lysates (*S. tm* ^ΔO^; *L. reuteri*; *E. faecalis*; *S. ent* ^wt^; *E. coli*; *S. tm* ^att^; *S. tm* ^att^ digested with proteinase K; *S. tm* M933 [no flagella, no functional TTSS]; flagellin FliC). Ig was detected with the respective *HRP*-labeled secondary antibodies. The experiment is representative for 6 different animals.

Next, we wanted to address if mice that had experienced a primary *S. tm* ^att^ infection in our model developed an adaptive immune response that would protect against gut inflammation upon re-infection with the same pathogen. This would be a pre-requisite for functional analysis of antibody responses in pathogen clearance. Therefore, we extended the infection model as depicted in Fig. 2A (‘immunization-challenge’ protocol). Sm-treated mice were infected with *S. tm* ^att^ for 39 days as in the standard protocol ( = experimental group; mock-immunization = negative control). This allowed sufficient time for recovering from acute inflammation and the generation of a *S. tm*-specific adaptive immune response (Fig. 1C; see also Fig. 2C,D). At day 39, the mice were treated with ampicillin to transiently suppress the microbiota and eliminate any *S. tm* ^att^ which may have persisted in the gut. We then challenged the animals with wild type *S. typhimurium* (wt; ampicillin resistant; 200 cfu by gavage). In the mock-immunized mice, wt *S. typhimurium* efficiently colonized the gut and elicited acute intestinal inflammation within two days after challenge (Fig. 2B). In contrast, the *S. tm* ^att^-immunized mice were generally protected against wt *S. typhimurium*-inflicted disease (8/10 mice with cecal pathology score ≤3; p = 0.0099; Fig. 2B).

Bacterial surface structures and secreted proteins are dominant targets of adaptive immune responses [18], [25], [26], [27], [28], [29]. Therefore, we analyzed whether surface-protein or O-antigen specific immune responses might explain the protection of *S. tm* ^att^-immunized mice. No protection was observed against challenge with the NTS serotype *S. enteritidis* (*S. en* ^wt^), harboring a different LPS-O-antigen, or an O-antigen deficient isogenic *S. typhimurium* mutant (*S. tm* ^ΔO^; Δ*wbaP*; Table S1; p>0.05 vs. colitis in mock immunized controls). Thus, *S. tm* ^att^-immunized mice mounted an adaptive immune response which protected from mucosal disease on re-infection with the pathogen in an O-antigen-dependent way.

### *S. tm* ^att^ induces pathogen-specific sIgA

The exquisite O-antigen specificity of protection from a second round of inflammation suggested that adaptive immunity and particularly sIgA may be the crucial mechanism not only for preventing inflammation on re-infection, but also for clearing pathogens from the gut. Therefore, we determined the kinetics of the *Salmonella*-specific humoral immune response by measuring specific Ig via surface staining of live, intact bacteria by flow cytometric analysis (Fig. 2C). This assay accurately differentiates *S. tm* specific antibodies from antibodies directed against closely related species, such as *E. coli* [30] (Fig.S1A–C). *S. tm*-specific IgM, IgG and IgA were detectable in the serum as early as 7 days post immunization. By day 14, all mice secreted *S. tm* -surface-specific sIgA into the gut lumen. Mucosal sIgA responses were confirmed by immunohistochemistry (Fig.S2). *Salmonella* antigens targeted by this strong, specific humoral immune response were analyzed by Western blotting. The antibody response was indeed pathogen-specific, as *Lactobacillus reuteri* RR and *Enterococcus faecali*s, two commensals isolated from our mouse colony, were not recognized (Fig. 2D; Fig.S3). In analogy to the human infection (Fig.S4), the antibody response included sIgA recognizing the O-antigen of *S. tm* (protease resistant ladder-like bands in the Western blot; Fig. 2D), a highly repetitive sugar structure of the lipopolysaccharide (LPS), coating the surface of the pathogen. In contrast, the O-antigens from *S. enteritidis* and *E. coli*, which have a different sugar structure or LPS from the O-antigen deficient mutant *S. tm* ^ΔO^ were not recognized. In addition, antibodies to several prominent protein antigens were detected. Most of these protein antigens were conserved in different *Salmonella* and *E. coli* strains, but not in *L. reuteri* RR or *E. faecali*s.

It should be noted that acute mucosal inflammation seems necessary to elicit immune responses protecting from enterocolitis. It was also shown previously, that invasive *Salmonella* strains triggered more potent adaptive immune responses [31]. Mice not pretreated with sm before immunization (low antigen loads, no gut inflammation), sm-treated mice immunized with *S. tm* ^avir^ (high antigen loads, no gut inflammation) and parenterally immunized mice (*S. tm* ^att^ i.v.; systemic antigen loads, no gut inflammation) did not mount detectable levels of O-antigen-specific sIgA. None of the mice were protected against wild type *S. tm* (*S. tm* ^wt^) mediated enteropathogenesis (Fig.S5).

Overall, these data demonstrated that the LPS O-antigen was the dominant protective antigen and that mice mount a robust pathogen-specific sIgA response during the first round of infection. This is in line with earlier data from studies in the mouse typhoid fever model, in chicken and data from human patients [18], [25], [26], [27] (Fig.S4). However, from these first sets of experiments we could not conclude whether pathogen-specific sIgA was sufficient for *S. tm* clearance from the gut.

### sIgA is dispensable for pathogen clearance from the gut lumen

**Figure 3:**
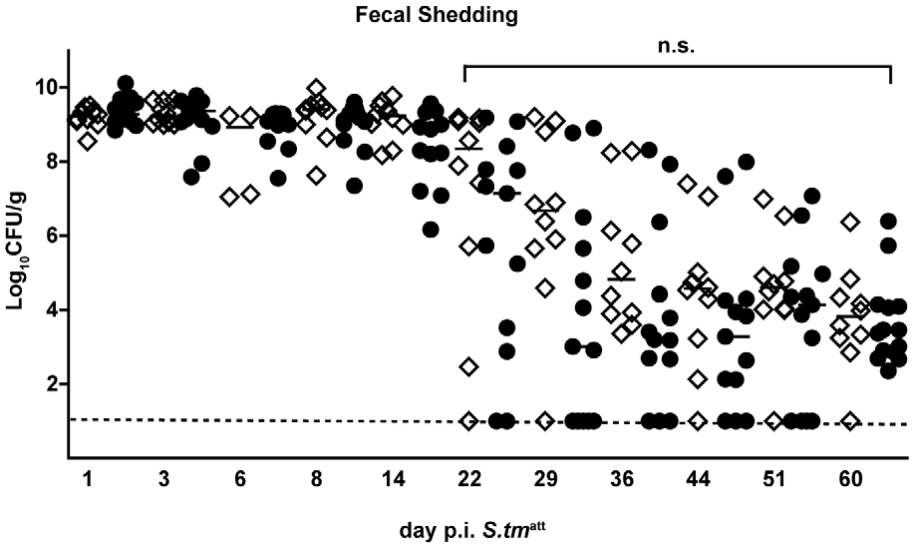
IgA deficiency does not affect kinetics of pathogen clearance. Time course of fecal *S. tm* ^att^ shedding in IgA^−/−^ and IgA-proficient littermates. IgA^−/−^ and IgA-proficient littermates were generated by crossing IgA^+/−^ mice in order to yield littermates with comparable gut flora. Sm-treated IgA^−/−^ (n = 10; white diamonds), IgA^+/−^ (n = 7; black circles) and wild type (n = 7; black circles) littermates were infected with *S. tm* ^att^ and fecal *Salmonella* shedding was monitored until day 60 post infection. Black bar: medians. Dashed line: detection limit. Statistics: comparison of fecal shedding by IgA^−/−^ vs. IgA^+/−^ and IgA^+/+^ (n.s.: p>0.05).

In order to address sIgA functions in pathogen clearance, we analyzed the outcome of *S. tm* ^att^ infection in different KO-mice lacking key mediators of functional adaptive immune responses. We determined whether T-cell dependent or -independent mucosal sIgA immune responses [30], [32], [33] were critical for termination of inflammation, pathogen clearance and protection from inflammation on re-infection. ‘Immunization-challenge’ experiments were performed on mice lacking the T-cell receptor (TCRβ^−/−^δ^−/−^; T-cell deficient), B-cells (J_H_^−/−^), IgA (IgA^−/−^) or sIgA and sIgM-transport into the gut lumen (pIgR^−/−^; Table S2). Two days after initial infection with *S. tm* ^att^, all knockout mice displayed pronounced gut inflammation (data not shown) and gut inflammation subsided by day 40 (Table 1). This demonstrated that the acute mucosal inflammation can be efficiently terminated in the absence of T-cells, B-cells, antibodies or sIgA. Furthermore, several IgA^−/−^ (3/4) and pIgR^−/−^ (2/5) animals managed to clear *S. tm* ^att^ from the gut lumen by day 40 p.i. This indicated that pathogens can (at least in some cases) be cleared from the gut lumen, in the absence of pathogen-specific sIgA (and sIgM) in the gut lumen. In order to exclude differences attributable to alterations in microbiota composition between different mouse lines, we have compared the *S. tm* ^att^ clearance kinetics between IgA^−/−^ and wild type littermates (IgA^+/−^, IgA^+/−^, IgA^+/+^; Fig. 3). This verified that kinetics of pathogen clearance was not affected by presence or absence of sIgA.

**Table 1:** Primary infection with *S. tm* ^att^.

Mouse line[Table-fn nt101] *	day 2	day 40						
	Gut inflam.‡	Cecum cont. (cfu/g)	MLN (cfu/organ)	Spleen (cfu/organ)	Gut inflam.‡	Antibodies		
						Serum IgG	Serum IgA	sIgA
wt	+	10	625	60	0	++	++	++
TCRβ^−/−^/δ^−/−^	+	6×10^7^	40	20	2	+#	+#	+#
J_H_^−/−¤^	+	2×10^3¤^	60¤	20¤	0¤	−¤	−¤	−¤
IgA^−/−^	+	10≠	1×10^3^	20	1	++	−	−
pIgR^−/−^	+	120§	1000	20	1	++	++	−

*median values of 4 or 5 mice per group; pathogen loads: cfu of *S. tm* ^att^.

**‡:** pathology score.

*median values of 4 or 5 mice per group.

# no LPS specific Ig.

**≠:** 3/4 IgA^−/−^ mice had no detectable *S. tm* ^att^ in the gut lumen.

**§:** 2/5 pIgR^−/−^ mice had no detectable *S. tm* ^att^ in the gut lumen.

**¤:** J_H_^−/−^ day 60 post *S. tm* ^att^ immunization.

Strikingly, none of the *S. tm* ^att^ -immunized knockout mice developed O-antigen specific antibodies and none were protected from intestinal inflammation upon challenge with *S. tm* ^wt^ (pathological score ≫3; Table 2 and Fig.S6). Thus, a T-cell dependent, adaptive mucosal sIgA response is essential for protection from secondary disease, but is dispensable for resolving the initial inflammatory response to *S. tm* ^att^ and for clearing the pathogen from the intestinal lumen.

**Table 2:** Challenge infection (day 2 post *S. tm* ^wt^ challenge).

Mouse line[Table-fn nt108] *	naïve mice				*S. tm* ^att^ immunized mice			
	Gut inflam.‡	Cecum cont. (cfu/g)	MLN (cfu/organ)	Spleen (cfu/organ)	Gut inflam.‡	Cecum cont. (cfu/g)	MLN (cfu/organ)	Spleen (cfu/organ)
wt	9	2×10^7^	4×10^3^	240	3	3×10^7^	7×10^2^	60
TCRβ^−/−^/δ^−/−^	10	1×10^7^	8×10^3^	290	8	4×10^8^	1×10^1^	280
J_H_^−/−^	9	2×10^7^	3×10^3^	20	8	5×10^8^	3×10^2^	20
IgA^−/−^	8	8×10^7^	4×10^3^	30	8	2×10^9^	1×10^3^	20
pIgR^−/−^	9	2×10^7^	2×10^3^	20	9	1×10^7^	2×10^3^	20

*median values of 4 or 5 mice per group; pathogen loads: cfu of *S. tm* ^wt^ (B).

**‡:** pathology score.

*median values of 4 or 5 mice per group.

**¤:** J_H_^−/−^ day 60 post *S. tm* ^att^ immunization.

### sIgA restricts intestinal pathogen growth and mucosal access

**Figure 4:**
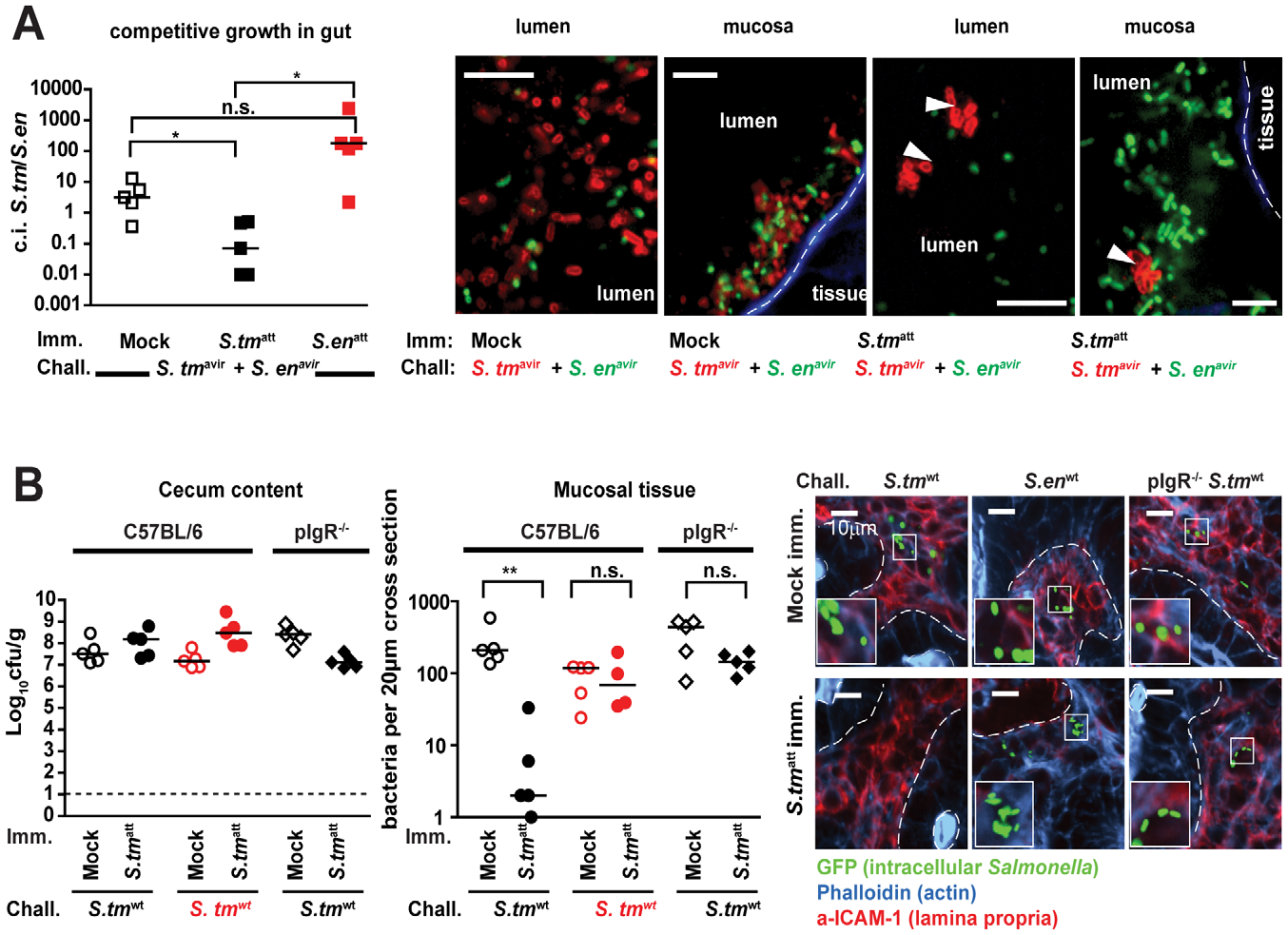
Immunization elicits O-antigen specific sIgA which retards pathogen growth and restricts mucosal access. **A**. sIgA retards pathogen growth and occludes mucosal access. Mice were immunized with *S. tm* ^att^, *S. en* ^att^ or mock (n = 5 per group), treated with ampicillin and challenged for 1 day with a 1∶1 mixture *S. tm* ^avir^(amp^R^; pWKS30) and *S. en* ^avir^ (amp^R^; pM979-GFP^+^). None of the mice developed gut inflammation. Left panel: competitive index (c.i.) of *S. tm* ^avir^(amp^R^; pWKS30) and *S. en* ^avir^ (amp^R^; pM979-GFP^+^) in the cecal lumen. Right panels: Immunofluorescence microscopy of the cecal lumen (left panels) and the mucosal surface (right panels; actin brush boarder = blue) to detect *S. tm* ^avir^ (red α-*S. tm* LPS stain) and *S. en* ^avir^ (green GFP^+^). Scale bar: 10µm. **B**. sIgA blocks mucosal invasion by *S. tm* ^wt^. C57Bl/6 (circles) or pIgR^−/−^ mice (diamonds) were immunized with mock (open symbols) or *S. tm* ^avir^ (closed symbols) and challenged with *S. tm* ^wt^ (black) or *S. en* ^wt^ (red). Bacterial loads in the cecal lumen (left) and *Salmonella* invasion into the mucosal tissue (middle) was determined at 2 days post challenge. Right panels: Immunofluorescence microscopy of GFP-expressing *S. tm* ^wt^ or *S. en* ^wt^ (green) in the cecal mucosa (red: ICAM-1, lamina propria; blue: Actin, epithelial brush border); White dotted lines mark epithelial-submucosal boarder; Scale bar: 10µm.

Though dispensable, our findings did not exclude that sIgA exerts an effector function [34] which contributes in some way to pathogen clearance. To identify such mechanisms, we analyzed the effects of sIgA on pathogen growth and its interaction with the host's intestinal mucosa in greater detail. First, we applied a modified ‘immunization-challenge’ protocol. Sm-treated mice were infected with *S. tm* ^att^, an equivalent *S. enteritidis* strain (*S. en* ^att^; *S. enteritidis* 125109 *sseD*; [35]) or mock. Antibody responses and *S. tm* ^att^/ *S. en* ^att^ loads in the stool were monitored (Fig.S7 and data not shown). After 39 days, immunized mice were treated with ampicillin (elimination of microbiota and remaining *S. tm* ^att^ or *S. en* ^att^) and challenged with a 1∶1 mixture of *S. tm^avir^* and *S. en^avir^* (ampicillin resistant; *sseDinvG* mutants; 200 cfu each by gavage; Table S1). These latter mutants can colonize the gut lumen of naïve mice for up to four days, remain confined to the gastrointestinal tract and they do not elicit enteropathogenesis, thus mimicking the situation in the intestines of ‘asymptomatic excretors’ [21], [24]. We decided not to use *S. tm* ^ΔO^ for this type of competition experiments as it displays a pronounced competitive growth defect in mice when co-infections are performed with an isogenic wild type strain [36]. In the gut lumen of *S. tm* ^att^ immunized mice, *S. en^avir^* out-competed *S. tm^avir^* (Fig. 4A; black symbols). In *S. en* ^att^ immunized mice, *S. tm^avir^* out-competed *S. en^avir^* (red symbols), and in mock-immunized mice, both strains colonized with equal efficiency. Therefore, O-antigen specific sIgA may help controlling pathogen growth or survival in the gut lumen. Furthermore, *S. tm^avir^* (but not *S. en^avir^*) was aggregated in the gut lumen and occluded from the mucosal surface of *S. tm* ^att^ immunized mice (Fig. 4A, right panels). Pathogen occlusion was confirmed by assessing pathogen loads in the gut tissue of challenged mice. In *S. tm* ^att^ -immunized animals, *S. tm* ^wt^ tissue loads were 100-fold lower than in mock-immunized controls (Fig. 4B). In contrast, *S. tm* ^att^ immunization did not prevent the invasion of *S. enteritidis*. Furthermore, *S. tm* ^att^ immunized pIgR^−/−^ mice, which cannot transport sIgA across the gut epithelium, failed to prevent gut tissue invasion by wt *S. typhimurium* into the mucosal tissue (Fig. 4B). Thus, the O-antigen-specific sIgA response conferred protection by restricting pathogen growth in the gut lumen and preventing the interaction of the pathogen with the intestinal mucosa. To some extent, this may also contribute to pathogen clearance from the gut lumen.

### Mice harboring a low-complexity microbiota become long-term asymptomatic excretors

While O-antigen-specific sIgA was indispensable to prevent disease, it did not seem to be a major determinant in pathogen clearance from the gut lumen. The onset of adaptive sIgA responses and cessation of symptoms seemed to occur well ahead of *S. tm* ^att^ elimination from the intestines. Moreover, IgA deficiency did not affect pathogen clearance kinetics (Table 1; Fig. 3). This was different from most well studied paradigms of acute systemic infection where the onset of protective immunity coincides with declining pathogen loads. This strongly suggested that sIgA-independent mechanisms may underlie pathogen clearance from the gut.

Thus, we hypothesized that the microbiota might play a crucial role in pathogen clearance. The microbiota is a dense bacterial community composed of approx. 500–1000 different species [9], [37]. It confers numerous beneficial effects to the host [38] including ‘colonization resistance’, i.e. a generalized interference with the growth of many pathogens in the gut of a naïve host [3]. Antibiotic treatment disrupts the normal microbiota, alleviates colonization resistance and constitutes a known risk factor for *Salmonella* infections in humans and mice [7], [9], [10], [21], [39]. Furthermore, the species composition of the microbiota - and by inference the degree of colonization resistance - can vary significantly between different individuals [40]. Therefore, the microbiota composition might explain why *Salmonella* shedding by ‘asymptomatic excretors’ can last for months or years.

In sm-treated mice, the microbiota is transiently reduced, but rapidly returns to pretreatment community composition, re-establishes ‘colonization resistance’ and ‘asymptomatic excretion’ occurs just transiently (Fig. 1C; [21], [41]). For this reason, our original infection model was not optimally suited for dissecting the differential role of the microbiota and sIgA in pathogen clearance.

**Figure 5:**
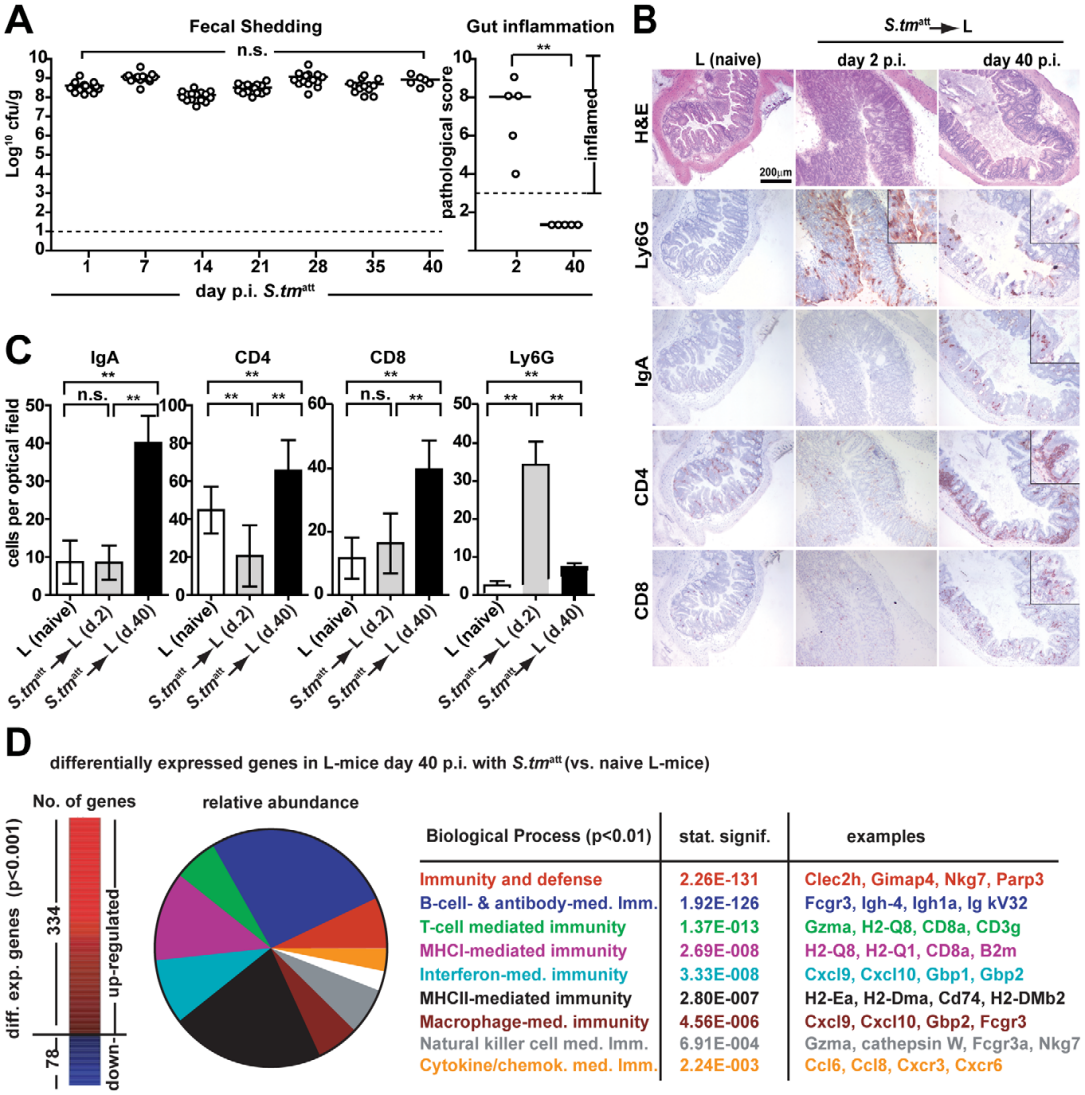
An O-antigen-specific sIgA response is insufficient to terminate *S. tm* ^att^ shedding by L-mice. **A**. L-mice do not clear *S. tm* ^att^ from the gut. L-mice (n = 15) were immunized with *S. tm* ^att^ (no streptomycin-treatment) and fecal shedding was monitored for 40 days (left panel). Gut inflammation was analyzed at day 2 and day 40 p.i. (5 mice per time point; right panel). **B. and C**. L-mice mount a pronounced adaptive mucosal immune response. Cecal tissue of naïve L-mice and L-mice at days two and 40 p.i. was stained by immunohistochemistry for Ly6G (granulocytes), IgA, CD4 (T-cells) and CD8 (T-cells) cellular markers. Quantitative data were from 15 randomly selected 40× high power fields (hpf) from 3–5 mice per group. Y-axis: average cell number per 40× hpf (**C**). **D**. Gene expression analysis of naïve and L-mice at day 40 post *S. tm* ^att^ immunization. Left: Numbers of differentially expressed genes in *S. tm* ^att^-immunized L-mice. Middle: relative abundance of biological pathways significantly upregulated in *S. tm* ^att^-immunized L-mice. Processes, p-values and characteristic examples are shown as a table (right).

**Figure 6:**
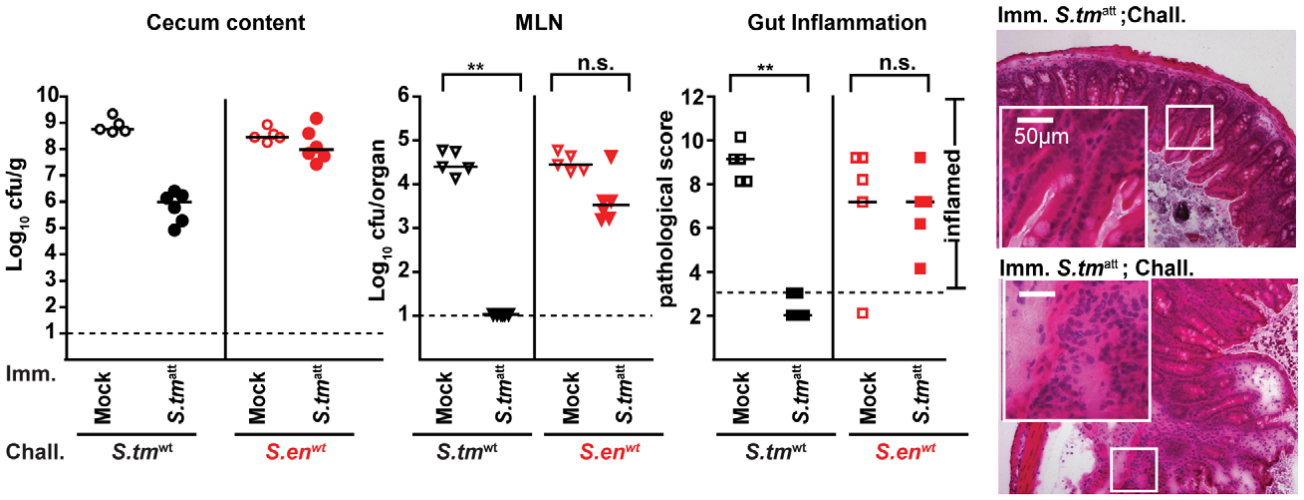
O-antigen specific protection from enteropathogenesis of *S. tm* ^att^-immunized L-mice. *S. tm* ^att^- (filled symbols) or mock- (open symbols) immunized mice were challenged for two days with *S. tm* ^wt^ (black; n = 10) or *S. en* ^wt^ (red circles; n = 5) at day 40 post immunization. Challenge-strain loads in the cecal content (left panel) and the MLN (middle panel) and cecal inflammation were assessed at day 2 post challenge. Dashed lines: detection limit or limit defining the absence of disease (pathological score ≤3). Black bars: median; *p<0.05; **p<0.005; n.s. = not significant. Right: H&E stained cross-sections of the cecum of *S. tm* ^att^-immunized L-mice at day two post challenge with *S. tm* ^wt^ (upper panel) or *S. en* ^wt^ (lower panel). Scale bar: 50µm.

To overcome this problem we used ‘L-mice’ which harbor a well defined, low complexity microbiota (L = ‘LCM mice’; [20]). L -mice are ex-germ free mice that are stably associated with the ‘Altered Schaedler Flora’ [42] comprising <20 species. The representatives with the highest abundance are ASF500 (Firmicutes; Clostridia; Clostridiales; Lachnospiraceae; unclassified_Lachnospiraceae) and ASF519 (Bacteroidetes; Bacteroidia; Bacteroidales; Porphyromonadaceae; Parabacteroides). Thus, the L microbiota resembles the conventional (C) microbiota of mice and men at broad lineages levels [43]. However, in spite of an equally high bacterial density as the C microbiota, the L microbiota does not confer colonization resistance [20]. Accordingly, *S. tm* ^att^ efficiently colonized the gut lumen of L-mice at high levels (≥10^8^cfu/g) and elicited pronounced enteropathogenesis by day 2 p.i. even without previous antibiotic treatment (Fig. 5A). After 40 days, all immunized L-mice had resolved acute inflammation, but kept on shedding *S. tm* ^att^ at high levels for at least 83 days (Fig. 5A; see also below). This was not due to a defective O-antigen-specific sIgA response: sIgA responses in L-mice were as pronounced as in C-mice as indicated by the increased numbers of IgA^+^ cells in the cecal mucosa (Fig. 5B,C and Fig.S2) and by Western Blot analysis (Fig.S8 and Fig 2D). The strong adaptive mucosal immune response was also confirmed by gene expression profiling of the cecal mucosa (Fig. 5D, Fig.S9, Table S3). Furthermore, challenge experiments confirmed the O-antigen-specific protection from enteropathogenesis (Fig. 6). However, despite this O-antigen-specific sIgA response, high-level pathogen shedding persisted in all analyzed animals (Fig. 5A; see also below). Therefore, O-antigen-specific sIgA was insufficient for luminal *S. tm* ^att^ clearance. This was in line with our hypothesis that elements of the normal, complex microbiota (which is lacking in L-mice) may play a key role in terminating fecal *S. tm* ^att^ shedding.

### Transfer of a complex microbiota facilitates pathogen clearance

**Figure 7:**
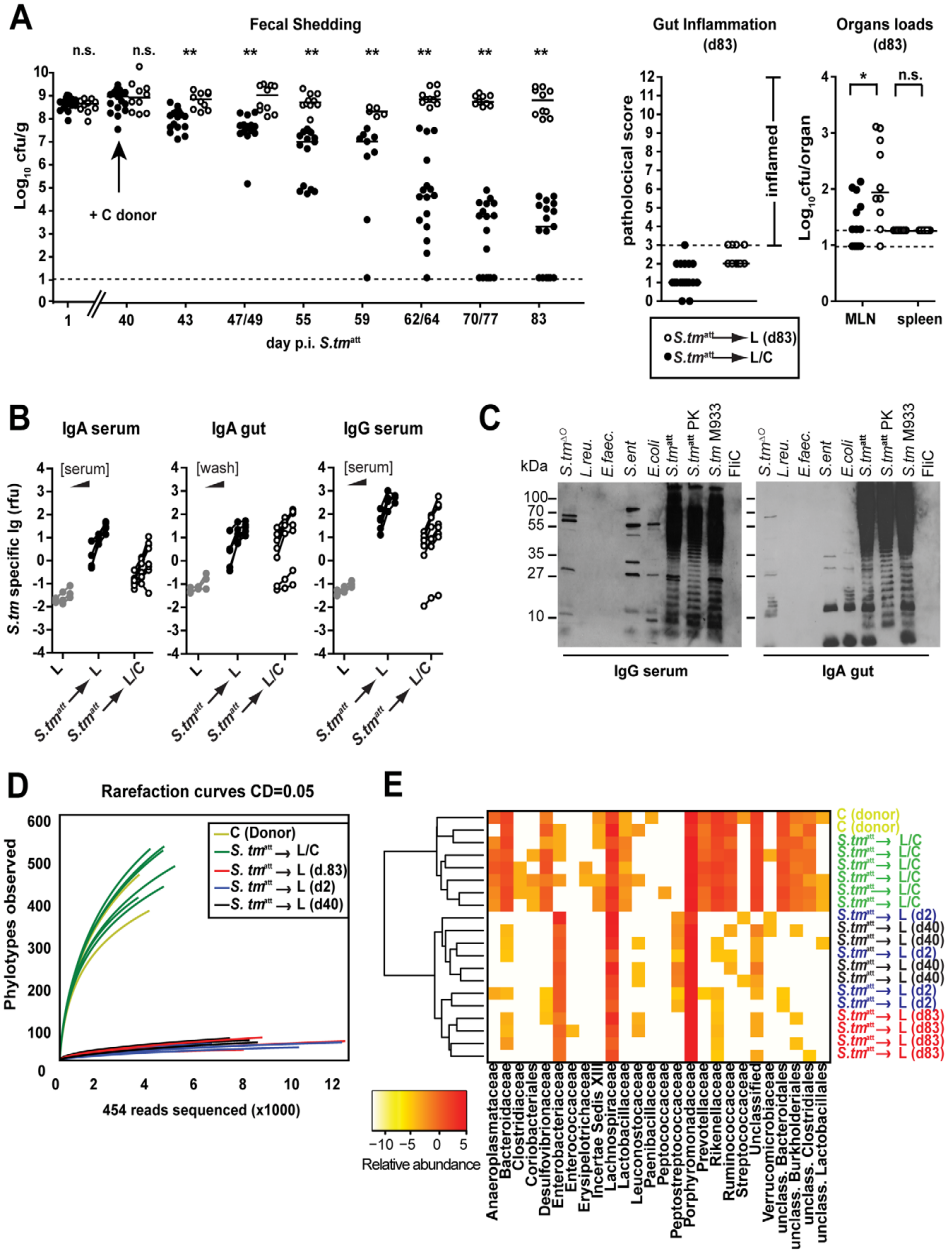
Transfer of a complex microbiota curbs *S. tm* ^att^ shedding by L-mice. **A**. Exposure to conventional gut microbiota curbs pathogen shedding by ‘asymptomatic excretors’. L-mice were immunized with *S. tm* ^att^. At day 40 p.i., donor animals with a conventional microbiota (C-mice) were added to one group (black circles; n = 17; *S. tm* ^att^→L/C). The other group remained under hygienic isolation (open circles; n = 10; *S. tm* ^att^→L). Fecal *S. tm* ^att^ shedding was monitored until day 83 p.i. Right panels show gut inflammation and organ loads at day 83 p.i.. **B. and C**. *S. tm*-specific Ig-response (serum IgA and IgG; IgA in gut wash; day 83 p.i.) in *S. tm* ^att^→L and *S. tm* ^att^→L/C mice was determined by bacterial FACS and by immunoblot as in Fig. 2A,B. **D**. A complex microbiota is transferred to L-mice by day 83 p. *S. tm* ^att^ immunization. Collectors' curves (CD = 0.05) were created for each mouse from the total number of filtered sequences for representative mice: *S. tm* ^att^→L (d.2), *S. tm* ^att^→L (d.40), *S. tm* ^att^→L (d.83), *S. tm* ^att^→L/C and 2 C-mice (donor). **E**. Phylotypes of mice shown in (**D**) (Clustering distance CD = 0.05) were sorted according to their family taxon (family level; x-axis) and average clustering was performed on Euclidean distances calculated between abundance profiles for each mouse and every time-point sampled. Red color indicates high abundance (Log2), yellow color low abundance.

To formally define the importance of the commensal microbiota in pathogen clearance, two groups of L-mice were infected with *S. tm* ^att^ for 83 days. The first group was kept under strict hygiene isolation and shed high loads of *S. tm* ^att^ until the end of the experiment (*S. tm* ^att^→L; Fig. 7A; open symbols). The second group was exposed to C microbiota at day 40 by placing C donor mice into the same cage (*S. tm* ^att^→L/C; 6 independent cages). Both groups of mice developed the typical pathogen-specific, adaptive sIgA response by day 83 p.i. (Fig. 7B,C). Upon introduction of the C donor mice, fecal shedding decreased gradually and ceased in most of the *S. tm* ^att^→L/C mice by day 83 (<10^5^cfu/g; Fig. 7A; black symbols) but not in the *S. tm* ^att^→L group. This suggested that pathogen clearance was mediated in some way by the complex microbiota.

In order to verify microbiota-transfer, we assessed microbiota composition using high-throughput 16S rRNA gene sequence analysis (Materials and Methods). *S. tm* ^att^→L/C mice displayed significantly higher diversity than *S. tm* ^att^→L mice as well as LCM mice at day 2 and 40 after *S. tm* ^att^ immunization (Fig. 7D). The rarefaction curves indicated that *S. tm* ^att^→L/C mice had acquired a microbiota of the similar complexity as the C donor mice. This was confirmed by assessing the richness (actual diversity) of the samples by calculating the Shannon index (H) and species evenness (E) as well as the Chao1 diversity estimate (Table S4). Accordingly, the taxonomy assignment confirmed that the number of bacterial taxa in the stool increased significantly in *S. tm* ^att^→L/C mice. All *S. tm* ^att^→L mice carried high loads of *Enterobacteriaceae* (i.e. *Salmonella* spp., *E. coli* spp.; red colors) in their stools. In contrast, no *Enterobacteriaceae* were detected in the stools of 3 (out of 6) *S. tm* ^att^→L/C mice and the remaining 3 animals carried low levels of this family (yellow colors, Fig. 7E). In addition, the microbiota composition was similar between all *S. tm* ^att^→L/C animals as demonstrated by hierarchical cluster analysis of eubacterial family profiles (Fig. 7E). This indicated that pathogen displacement occurs in a reproducible, stereotypic fashion and may not result from random transfer of only few members of the conventional microbiota. Most importantly, these data demonstrate that members of the conventional microbiota can upon transfer lead to the termination of sustained pathogen shedding in L-mice.

**Figure 8:**
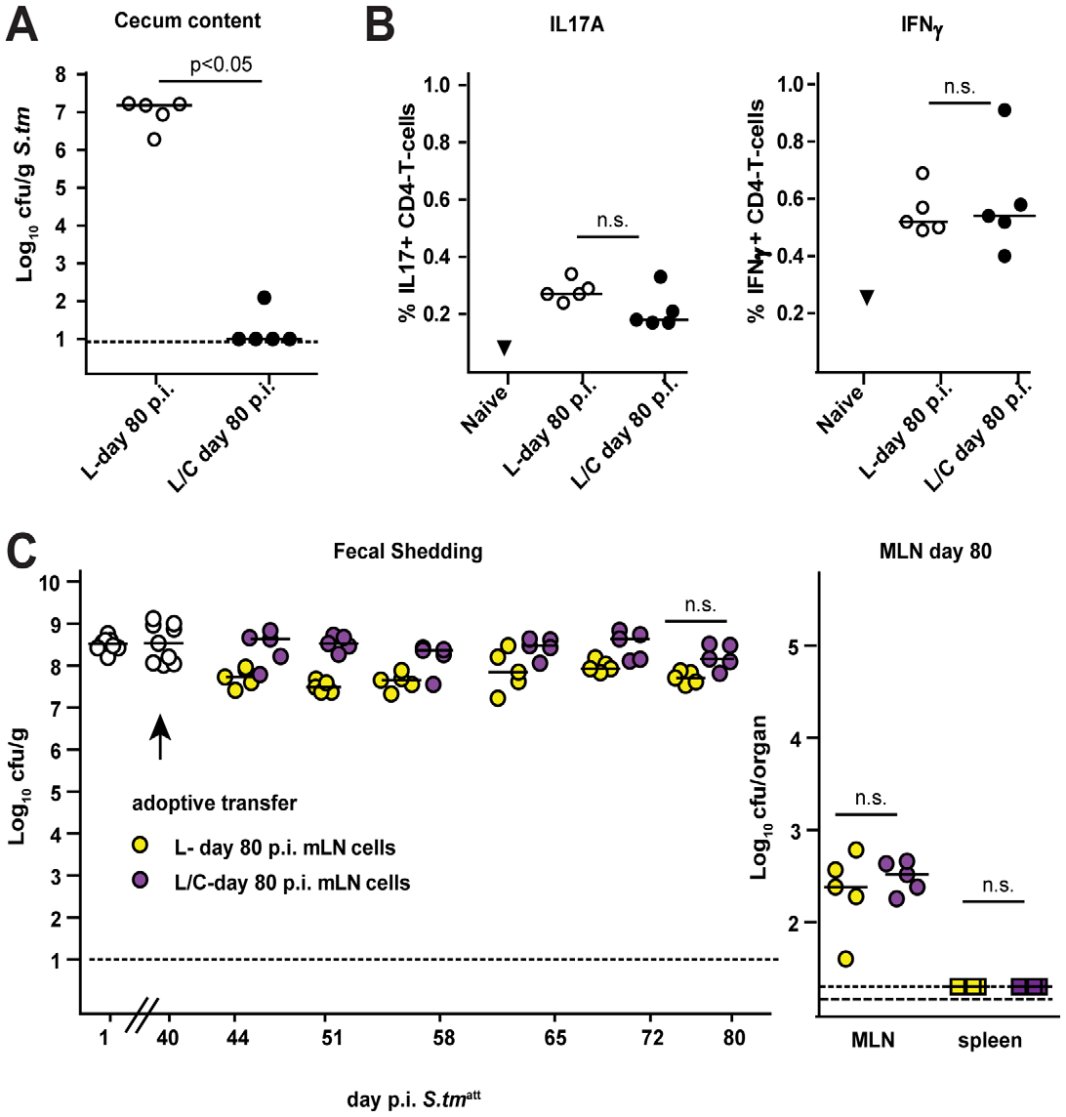
Adoptive transfer of MLN cells isolated form mice that cleared *S. tm* ^att^ from the intestine into *S. tm* ^att^→L mice (day 40 p.i.) does not induce *S. tm* ^att^ clearance. L-mice were immunized with *S. tm* ^att^. At day 40 p.i., animals with a conventional microbiota (C-mice) were added to one group (black circles; n = 5; *S. tm* ^att^→L/C). The other group remained under hygienic isolation (open circles; n = 5; *S. tm* ^att^→L). **A**. All mice were sacrificed at day 80 p.i. and *S. tm* ^att^ levels in the cecal content were defined. **B**. Total MLN cells from the two groups of mice were isolated and analyzed with respect to the expression of intracellular cytokines IL-17A (left) and IFNγ (right) in CD4^+^ T-cells at day 80 post *S. tm* ^att^ infection (% cytokine-expressing CD4^+^ T-cells). **C**. 3×10^7^ MLN cells of ‘donor’ mice *S. tm* ^att^→L and *S. tm* ^att^→L/C described above were adoptively transferred into another cohort of *S. tm* ^att^→L mice (day 40 p.i.: = ‘aceptors’). Fecal *S. tm* ^att^ shedding was followed in the ‘acceptors’ until day 80 post *S. tm* ^att^ infection ( = day 40 post adoptive transfer, left). *S. tm* ^att^ levels in the MLN of ‘acceptors’ at day 80 post *S. tm* ^att^ infection ( = day 40 post adoptive transfer, left).

It remained unclear whether pathogen clearance was mediated directly by the microbiota or by microbiota-induced mucosal responses. Recently, it has been shown that parts of the microbiota (i.e. segmented filamentous bacteria) induce mucosal TH-17 cell responses that can protect from pathogen infection [2]. However, we did not observe differences in IL-17A or IFN gamma-producing CD4 T-cells in the MLN of *S. tm* ^att^→L and *S. tm* ^att^→L/C animals by day 83 (Fig. 8). Furthermore, we tested if total MLN cells obtained from *S. tm* ^att^→L/C animals would, upon transfer into *S. tm* ^att^→L (d.40) induce clearance of intestinal *S. tm* ^att^. However, this was not the case (Fig.8). This was in line with the notion that the microbiota may directly mediate pathogen clearance.

## Discussion

In this study, we have defined the contributions of sIgA and the microbiota in protecting the host from NTS infection. During the first encounter with the pathogen, the microbiota mediates at least two different protective functions, colonization resistance and pathogen clearance. The former is well established and prohibits the growth of diverse incoming pathogens, thus preventing colonization right on [3], [5], [20], [44]. Here, we identified pathogen clearance as a second protective function attributable to the microbiota. Pathogen clearance eliminates the pathogen from the gut lumen after an episode of acute infection, i.e. after *Salmonella* diarrhea. This differs from colonization resistance as the pathogen starts out at high density and the normal microbiota must re-grow from a state of depletion and disturbed composition (i.e. caused by the pathogen and the inflammatory response). Compared to the microbiota, an adaptive sIgA response mounted during the later stages of an acute infection contributes little to clearing the pathogen from the gut lumen. However, pathogen-specific sIgA protects from mucosal inflammation if the same pathogen is encountered for a second time. Thus, the microbiota and sIgA have complementary functions which jointly protect against enteropathogenic bacteria during the initial infection and subsequent exposure.

How does the microbiota mediate pathogen clearance? The lack of suitable assay systems has hampered addressing this question in the past. Clearly, *S. tm* clearance starts out in a situation where the pathogen has grown up in the gut lumen, inflicted disease and thereby slashed microbiota density, composition and/or function [45]. This situation is gradually reversed, involving the decrease of luminal pathogen loads as well as microbiota re-growth. Finally, normal microbiota composition, density and function are restored. Conceivably, some of the mechanisms conferring colonization resistance, i.e. bacteriocin production, inhibitory metabolites, oxygen depletion, receptor blocking, stimulating mucin- or antimicrobial peptide release, stabilization of the mucosal barrier, improvement of gut motility and/or nutrient limitation [5], might also contribute to different phases of pathogen clearance. Also, microbiota-mediated stimulation of the mucosal cellular immune system may be involved [2], [46] even though TH-17 mediated responses do not seem to contribute significantly to *S. tm* clearance, as indicated by adoptive transfer experiments (Fig. 8). The mechanisms mediating clearance and the relative importance of the microbiota, sIgA and other mucosal immune responses may differ between different pathogens or even between different strains of a given pathogen. Identifying the commensal species (or consortia) involved and the molecular mechanisms mediating pathogen clearance will be an important task for future research.

Does sIgA contribute to pathogen clearance? Pathogen specific sIgA is produced by wild type animals during the phase of pathogen clearance. O-antigen-specific sIgA led to aggregation of luminal pathogens, prevented access to the enterocyte surface and reduced net pathogen growth as indicated by a reduced competitive index. Surprisingly, this had little effect on *S. tm* clearance. Wild type mice and IgA^−/−^ littermates displayed equivalent rates of pathogen clearance. Moreover, sIgA did not reduce pathogen loads in the stool, at least in the L-mice in the absence of a complex gut flora. Thus, for pathogen clearance at the end of a primary enteric *S. tm* infection, a pathogen-specific sIgA response is neither necessary nor sufficient.

Instead, sIgA protected from mucosal inflammation upon re-infection with the same pathogen. The LPS O-antigen was the key protective antigen of this adaptive immune response. Protection was attributable to pathogen-aggregation in the gut lumen, reduced net pathogen growth and pathogen-exclusion from the epithelial surface, thus inhibiting pathogen invasion into the gut tissue. This required sIgA transport into the gut lumen, as immunized pIgR^−/−^ mice, which fail to transport sIgA across the intestinal epithelium, have equivalent pathogen loads in the gut mucosa as non-immunized littermates or wild type animals. Thus, pathogen specific antibodies do not seem to contribute much to protection, once *S. tm* has breached the mucosal barrier. This is in line with earlier work on the roles of antibody responses in systemic *S. tm* infection [17], [47]. In conclusion, our experiments show that O-antigen-specific sIgA responses protect against *Salmonella*-mediated gut inflammation upon re-infection.

It is interesting to consider the protective function of sIgA and the microbiota from an evolutionary perspective. The intestines of most animals are colonized by bacterial communities [43]. It seems safe to assume that microbiota have an evolutionary ancient function in protecting from infection. We speculate that this pertains to colonization resistance and to pathogen clearance and that both have evolved to provide protection against a broad range of pathogens. The elaborate adaptive immune system of modern mammals, including sIgA responses, evolved much later. It evolved in the presence of the protective functions provided by the microbiota, i.e. colonization resistance and pathogen clearance. The high efficiency of this microbiota-mediated protection may explain why sIgA responses have not evolved to affect the first round of infection with a given pathogen. This was simply not necessary. In contrast, evolving the sIgA response to protect in the case of repeated exposure to the same pathogen may have represented a strong benefit which cannot be accomplished by the microbiota. This evolutionary history may explain why the sIgA response contributes little during the primary infection. Anyhow, in modern mammals the microbiota and sIgA have quite different protective functions which complement each other during the initial- and subsequent encounters with a given pathogen.

An ‘unfavorable’ microbiota composition, e.g. in L-mice, can result in long term shedding. Asymptomatic NTS excretion is also observed in humans recovering from acute diarrhea. This period of asymptomatic excretion normally lasts for two to eight weeks, but may last for more than a year in a few patients. This poses a risk of transmission. In analogy to the long term shedding by L-mice, we propose that these individuals might lack some unidentified component of the normal microbiota. In L-mice, the pathogen is cleared upon transferring microbiota from a healthy donor. This may have implications for managing human long term asymptomatic excretors. Traditionally, patients are advised to adhere to strict personal hygiene and might even be isolated in order to reduce the risk of transmission. However, at the same time this deprives the patients from exposure to conventional microbiota from healthy individuals which might enhance pathogen clearance. So far, we do not know the species of the microbiota, the cellular interactions, and molecular mechanisms explaining pathogen clearance. However, the experimental systems presented in our study may provide the tools to address these important issues. Our findings provide a basis for future research on optimal management of ‘asymptomatic excretors’, NTS vaccine development and microbiota-directed therapy for acute diarrheal NTS infections.

## Materials and Methods

### Animals

Specified pathogen-free (SPF) wild type C57BL/6 mice, J_H_^−/−^ [48] and IgA^−/−^ [49], pIgR^−/−^ [50] and TCRβ^−/−^δ^−/−^ mice [51] (7–10 weeks old; all C57BL/6 background) were bred at the Rodent center HCI (RCHCI) under barrier conditions in individually ventilated cages (Ehret). IgA^−/−^, IgA^+/−^ and IgA^+/+^ littermates were generated by crossing IgA^−/−^ with C57BL/6 mice and breeding IgA^+/−^×IgA^+/−^ animals. L-mice were generated by colonizing germfree C57BL/6 mice with the Altered Schaedler flora (ASF). Mice, housed in a bubble isolator, were inoculated at eight weeks of age by intra-gastric and intra-rectal administration of 10^7^–10^8^ cfu of ASF bacteria on consecutive days (www.taconic.com/library). Later, L-mice (C57BL/6 background) were maintained under barrier conditions in IVCs with autoclaved chow and autoclaved, acidified water. Mice with complex microbiota were never housed together with these in the same room to prevent contamination with additional commensal bacteria.

### Ethics statement

All animal experiments were approved (license 201/2004 and 201/2007 Kantonales Veterinäramt Zürich) and performed according to local guidelines (TschV, Zurich) and the Swiss animal protection law (TschG).

### Infection experiments

*Salmonella* infections were performed in individually ventilated cages at the RCHCI, Zurich, as previously described [52]. In brief, wild type C57BL/6 mice, J_H_^−/−^, IgA^−/−^, pIgR^−/−^ and TCRβ^−/−^δ^−/−^ mice were pretreated with 20mg of streptomycin (sm) by gavage. 24h later, the mice were inoculated with 5×10^7^ CFU of *S. tm* ^att^, PBS (mock) or as indicated. ‘Challenge infections’ were performed 40 days later (or as indicated). Mice were treated with ampicillin (20mg; by gavage) and 24h later infected with a dose of 200 CFU of the respective ampicillin-resistant (pM973) challenge strain. Samples of cecal tissue were cryo-embedded, and inflammation was quantified on cryosections (5 µm, cross-sectional) stained with hematoxylin and eosin (H&E). Pathogen-colonization was assessed as described, below.

### Histology

H&E-stained cecum cryosections were scored as described, evaluating submucosal edema, PMN infiltration, goblet cells and epithelial damage yielding a total score of 0–13 points [53].

### Analysis of *Salmonella* loads in cecal content, mesenteric lymph nodes, and spleen

Mesenteric lymph nodes (MLN), spleen and liver were removed aseptically and homogenized in cold PBS (0.5% tergitol, 0.5% BSA). The cecum content was suspended in 500ìl cold PBS and bacterial loads were determined by plating on MacConkey agar plates (50 ìg ml^−1^ streptomycin) as described [21]. Colonization levels of the challenge strain (carrying pM973 with an antibiotic marker) and immunization strain (*S. tm* ^att^; km^R^) were determined by selective plating (100ìg ml^−1^ ampicillin or 30 ìg ml^−1^ kanamycin; levels of challenge strain: amp^R^-km^R^ *Salmonella*). For co-infection experiments shown in Fig. 2C, competitive indices were calculated according to the formula CI = ratio *S. tm*:*S. en*_cecal content_/ratio *S. tm*:*S. en*_inoculum_.

### Adoptive transfer of MLN cells

MLN were harvested from *S. tm* ^att^→L (total of 5 mice) and *S. tm* ^att^→L/C (total of 5 mice) at day 80 post *S. tm* ^att^ infection. Single cell suspensions were prepared using 100µm cell strainers and 40µg/ml DNAse (Roche). *S. tm* ^att^→L mice (at day 40 post *S. tm* ^att^ infection) were injected intravenously with 3×10^7^ cells (pooled for each of the two groups) in 200µl PBS. Fecal *S. tm* ^att^ shedding was monitored for another 40 days.

### Intracellular cytokine staining

Single-MLN-cell suspensions were prepared as described above. For intracellular staining of IFN-γ and IL-17A, 1×10^7^ nucleated MLN cells were cultured for 3 h in 1 ml of RPMI 1680 supplemented with 10% heat-inactivated FCS and stimulated with PMA (5pg/ml) /ionomycin (500pg/ml). After adding 20 µg/ml brefeldin A, the cells were incubated for another 3h at 37°C. Cells were harvested and washed in ice-cold FACS buffer (PBS, 2% heat-inactivated FCS, 5 mM EDTA, and 0.02% sodium azide). Cells were resuspended in FACS buffer and stained on the surface with fluorescently-labeled antibodies for 30 min on ice. For intracellular staining of IL-17A and IFN-γ, cells were washed once and fixed/permeabilized for 10 min at room temperature using 500 µl of FIX/perm solution (FACSLyse; BD Biosciences; diluted to 2× concentration in distilled water and 0.05% Tween 20). Cells were washed once and stained with directly conjugated Abs against IFN-γ-APC (BD) and IL-17A-PE (Biolegend). Cells were then washed again and resuspended in PBS. Data were collected on a LSRII flow cytometer (BD Biosciences) and analyzed using FlowJo software (Tree Star).

### Gut wash

The intestine was flushed with 2 ml of a washing buffer containing PBS, 0.05M EDTA pH8.0 and 66µM PMSF. Intestinal wash was briefly vortexed and centrifuged at 4°C, 30 min, 40.000 rpm (Eppendorf centrifuge). Aliquots of supernatants were stored at −80°C.

### Immunofluorescence microscopy

Bacteria harboring pM973 (GFP expression after tissue entry) in the lamina propria and epithelium were enumerated by fluorescence microscopy as described [22] using cryo-sections of PFA-fixed cecal tissue stained with Armenian hamster anti-CD54 (clone 3E2; stains lamina propria) antibody (Becton Dickinson), Cy3-conjugated goat anti–Armenian hamster Ig (Jackson ImmunoResearch Laboratories), DAPI (stains DNA; Sigma-Aldrich), and Alexa647-conjugated phalloidin (stains polymerized actin; Fluoprobes). We evaluated three 20 µm thick sections of the cecum per mouse and plotted for each mouse the average of the three values.

For detecting *S. tm* and *S. en* pM979 in the gut lumen *in situ*, cecal tissues were recovered and treated as described recently [54]. Briefly, the tissues were fixed in paraformaldehyde (4% in PBS, pH 7.4 over night, 4°C), washed with PBS, equilibrated in PBS (20% sucrose, 0.1% NaN_3_ over night, 4°C), embedded in O.C.T. (Sakura, Torrance, CA), snap-frozen in liquid nitrogen and stored at −80°C. Cryosections (7ìm) were air-dried for 2 h at room temperature, fixed in 4% paraformaldehyde (5 min), washed and blocked in 10% (w/v) normal goat serum in PBS for 1h. *S. tm* was detected by staining for 1h with a polyclonal rabbit á-*Salmonella*-O-antigen group B serum (factors 1, 4, 5 and 12, Difco; 1∶500 in PBS, 10% (w/v) goat serum) and Cy3-conjugated secondary goat-α-rabbit antibody. *S. en* pM979 expresses *gfp* under the control of a constitutive promoter and bacteria were detected in the green channel. F-Actin (epithelial brush border) was visualized by staining with Alexa-647-conjugated phalloidin, as indicated (Molecular Probes). Sections were mounted with Vectashield hard set (Vector laboratories) and sealed with nail polish. Images were recorded with a microscope (Axiovert 200; Carl Zeiss, Inc.), an Ultraview confocal head (PerkinElmer), and a krypton argon laser (643-RYB-A01; Melles Griot). Infrared, red, and green fluorescence was recorded confocally, and blue fluorescence was recorded by epifluorescence microscopy.

### Immunohistochemistry

Frozen consecutive sections of spleen, liver, cecum, colon and small intestine (7ìm thick) were briefly fixed (10 min) in acetone and blocked for 30 min with phosphate buffered saline (PBS) containing 0.5% bovine serum albumin (BSA). Sections were then incubated with the primary antibody for 1 h at room temperature. Primary antibodies included: B220/CD45R (Pharmingen 553084; 1∶200) , CD4 (clone YTS191; 1∶200) and CD8 (clone YTS169; 1∶200) for T-cells (kindly provided by Rolf Zinkernagel; 1∶50), Ly-6G (Gr-1) for neutrophils (clone RB6-8C5; 1∶600), F4/80 for macrophages (Serotec MCAP 497; 1∶50), CD11c for dendritic cells (BD Biosciences 553800; 1∶100) and IgA (rat-anti-mouse IgA; Pharmingen 556969; clone C10-3; 1∶4000). Secondary antibodies and detection chromagens were applied and visualized using standard methods (see also [55]).

### Statistical analysis

Statistical analysis was performed using the exact Mann-Whitney U test (Prism 4.0c). A *P* value of <0.05 (two tailed) was considered to be statistically significant. In mouse experiments, values were set to the minimal detectable value (10 cfu for cecum; 10 CFU for MLNs; 20 CFU for the spleen) for samples harboring “no bacteria.” Two figures (Fig. 1A and 4F) were generated using the statistical software package R. To assess the distribution of *Salmonella* loads in mice during the 60 day infection experiments, median and quantiles (corresponding to 0.05, 0.25, 0.75 and 0.95 probabilities) were plotted for each day or group of days. We performed a linear regression on medians and both 0.05 and 0.95 quantiles, weighted by the number of data points sampled for each day.

The OTUs abundance heatmap represents the mouse normalized OTU abundances (log2) clustered by average linkage clustering on Euclidean distances. This was generated using the function ‘heatmap.2’ from the ‘gplots’ R library.

### Immunoblot analysis

The equivalent of 1 OD_600_ units/ml (where OD_600_ is the optical density at 600nm) of liquid o.n. cultures of *S. tm* ^ΔO^, *Lactobacillus reuteri* RR, *Enterococcus faecalis*, *S. en* ^wt^, *E.coli*, *S. tm* ^wt^, *S. tm* ^wt^ proteinase K treated (Gibco/Life Technologies; 0.4 mg/ml; 1h 57°C) or *S. tm* M933 was pelleted by centrifugation at 14,000×*g* for 2 min, and the supernatant was discarded. Cells were resuspended in Laemmli sample buffer (0.065 M Tris-HCl [pH 6.8], 2% [wt/vol] sodium dodecyl sulfate [SDS], 5% [vol/vol] β-mercaptoethanol, 10% [vol/vol] glycerol, 0.05% [wt/vol] bromophenol blue) and lysed for 5 min at 95°C. Equal amounts of the different strains and purified *S. tm* flagellin FliC were loaded on a 12% SDS-polyacrylamide gel and proteins wer separated by electrophoresis. Immunoblots were stained with mouse serum (diluted 1∶200 in PBS) or intestinal lavages (diluted 1∶20 in PBS) from naïve or immunized mice, goat-α-mouse-IgA HRP (Southern Biotech), goat-anti-mouse-IgG HRP (Bethyl Laboratories) and developed using an ECL kit (Amersham). The same protocol was used for the analysis of the human patient serum (dilution 1∶20 in PBS), where goat-anti-human-IgA-HRP (2050-05, NEB) and goat-anti-human-IgG-HRP (2040-05, NEB) were used as secondary antibodies.

### Bacterial FACS

Analysis was performed as described in [30]. 3ml LB cultures were inoculated from single colonies of plated bacteria and cultured overnight at 37°C without shaking. 1ml of culture was gently pelleted for 4min at 7,000 rpm in an Eppendorf minifuge and washed 3× with sterile-filtered PBS (1% BSA, 0.05% sodium azide) before resuspending to yield a final density of 10^7^ bacteria per ml. Mouse serum was diluted 1∶20 in PBS (1% BSA, 0.05% sodium azide) and heat-inactivated at 60°C for 30min. The serum solution was then spun at 13,000 rpm in an Eppendorf minifuge for 10min to remove any bacteria-sized contaminants and the supernatant was used to perform serial dilutions (1∶20, 1∶60, 1∶180). 25µl serum solution and 25µl bacterial suspension were mixed and incubated at 4°C for 1h. Bacteria were washed twice before resuspending in monoclonal FITC-anti-mouse IgA (559354; BD Pharmingen), PE-anti-mouse total IgG (715-116-151; Jackson Immunoresearch Europe) and APC-anti-mouse IgM (550676; BD Pharmingen). After a further hour of incubation bacteria were washed once with PBS (1% BSA, 0.05% sodium azide) and then resuspended in PBS (2% PFA) for acquisition on a FACSCalibur using FSC and SSC parameters in logarithmic mode. Data were analysed using FlowJo software (Treestar, USA). Analysis of specific IgA in intestinal lavages was achieved using an identical protocol, using a dilution of 1∶2, 1∶6 and 1∶18 of gut wash.

### Microarray-Analysis

The cecum tissue was excised (3 biological replicates per group), washed in cold PBS, placed in 300µl RLT-buffer (RNeasy Mini Kit, Qiagen; 1% β-Mercaptoethanol) and snap-frozen in liquid nitrogen. Total RNA was extracted with the Nucleospin RNA II kit (Macherey Nagel, Germany) and prepared for hybridization as recommended by the manufacturer (Applied biosystems, USA). Briefly, 2µg of total RNA and a T7-oligo(dT) primer were used for reverse transcription. The double-stranded cDNA was purified and converted to DIG labeled-cRNA by in vitro transcription using DIG-UTP (Roche, Germany). The cRNA was purified, fragmented and hybridized on ABI Mouse Genome Survey v2.0 microarrays for 16h. The microarray was washed and incubated with anti-DIG antibodies conjugated to alkaline phosphatase and a chemiluminescent substrate. The microarrays were scanned with the Applied Biosystems 1700 chemiluminescent microarray analyzer [56]. Normalization was achieved using the NeONORM method [57]. Significance of log_2_ fold changes (log_2_Q) were determined based on a double-log normal distribution hypothesis of signal intensities using mixture ANOVA methodology [56]. A change in the gene expression profiles was considered as significant if *p*<0.001. Heat maps were created according to standard methods [56]. Gene Ontology (GO) annotations were analyzed using the Panther Protein Classification System (http://www.pantherdb.org). Microarray data were deposited in the publicly available database: http://mace.ihes.fr with accession number: 2947924142.

### Bacterial DNA extraction and 16SrRNA gene specific PCR

Total DNA was extracted from cecal contents using a QIAmp DNA stool mini kit (Qiagen). Bacterial lysis was enhanced using 0.1mm glass beads in buffer ASF and a Tissuelyzer device (5 minutes, 30Hz; Qiagen). V5-V6 regions of bacterial 16S rRNA were amplified using primers B-V5 (5′ GCCTTGCCAGCCCGCTCAG ATT AGA TAC CCY GGT AGT CC 3′) and A-V6-TAGC (5′GCCTCCCTCGCGCCATCAG [TAGC] ACGAGCTGACGACARCCATG 3′). The brackets contain one of the 20 different 4-mer tag identifiers [TAGC, TCGA, TCGC, TAGA, TGCA, ATCG, AGCT, AGCG, ATCT, ACGT, GATC, GCTA, GCTC, GATA, GTCA, CAGT, CTGA, CAGA, CTGT, CGTA]. Cycling condition were as follows: 95°C, 10min; 22 cycles of (94°C, 30s; 57°C, 30s; 72°C, 30s); 72°C, 8min; 4°C, ∞; Reaction conditions (50µl) were as follows: 50ng template DNA; 50 mM KCl, 10 mM Tris-HCl pH 8.3, 1.5 mM Mg^2+^, 0.2mM dNTPs; 40pmol of each primer, 5U of Taq DNA polymerase (Mastertaq; Eppendorf).

PCR products of different reactions were pooled, ethanol-precipitated and fragments of ∼300bp were purified by gel electrophoresis, excised and recovered using a gel-extraction kit (Machery-Nagel). Amplicon sequencing of the PCR products was performed using a 454 FLX instrument (70×70 Picotitre plate) according to the protocol recommended by the supplier (www.454.com). PCR to detect ASF bacteria in the feces was done as described in [42].

### Quality filtering and reads sorting

We applied quality control to 454 reads in order to avoid artificial inflation of ecosystem diversity estimates [58]. Reads containing the consensus sequence (‘ACGAGCTGACGACA[AG]CCATG’) of the V6 reverse primer were filtered with respect to their length (200nt≤length≤300nt). Quality filtering was then applied to include only sequences containing one of the exact 4nt tag sequences and displaying at maximum one ambiguous nucleotide ‘N’. The latter criterion has been reported as a good indicator of sequence quality for a single read [41]. We identified 6,754 reads with an incorrect primer sequence, 1,155 reads shorter than 200nt, 8 reads longer than 300nt and 119 reads containing more than one ‘N’. After filtering, 140,237 reads remained (out of an initial total of 149,786 raw reads) and were processed as described below for OTU definition and chimera filtering. To estimate the reliability of sample discrimination using our primer-tagging approach, we assessed the number of reads observed to have an illegitimate 4-mer tag (i.e., different from our set of 20 tags). The sequencing plate produced a total of 141,784 quality-filtered reads from which 1,547 contained an incorrect tag (1.09%). Given that 256 distinct 4-mer tags are possible and that we used only 20 of these, the majority of sequencing or primer errors in this region are detectable. Correcting for the small fraction of undetectable errors (20/256) and division by four yields an error rate of 0.296% per single nucleotide - at the position of the tag in the primer (this includes errors during primer synthesis as well as sequencing). Since most errors are actually visible as errors, the rate of unintentional ‘miscall’ of sample identity is 0.092%.

### Definition of OTUs

To reduce computational time and complexity, we built OTUs using the complete filtered dataset covering all non-redundant reads from the 20 samples. Exactly identical sequences were represented by one representative only; after OTU computation, redundant sequences were taken into account for OTU abundance analysis. For subsequent taxonomy classification, we included additional quality-filtered 16S rRNA reference sequences, selected from the Greengenes database (http://greengenes.lbl.gov/Download/Sequence_Data/Greengenes_format/greengenes16SrRNAgenes.txt.gz, release 01-28-2009 [59]). This reference database is based on full-length non-chimerical sequences with a minimum length of 1100nt (in order to fully cover the V6 region of all entries). No archaeal sequences were included in the analysis.

The alignment of non-redundant reads from all mice with the reference database was performed using the secondary-structure aware Infernal aligner (http://infernal.janelia.org/, release 1.0, [60]) and based on the 16S rRNA bacterial covariance model of the RDP database (http://rdp.cme.msu.edu/; [61]). Before defining OTUs, we first removed reference sequences for which the alignment was not successful (Infernal bit-score<0). The alignment was then processed to include an equivalent amount of information from every read. To do so, we identified the consensus reverse primer sequence of the V6 region within the aligned sequence of *Escherichia coli* K12, as a reference. The full alignment was then trimmed from the start position (defined by the *E. coli* V6 reverse primer) and ended after 200nt. This also ensured a further limitation of the effect of pyrosequencing errors by trimming the 3′ end of each read, a region which is more sequencing-error prone (the trimmed and aligned reads length ranged from 152 to 231nt) [58]. Using this alignment, OTUs were built by hierarchical cluster analysis at various distances (0.01, 0.03, 0.05 and 0.10) using the ‘complete linkage clustering’ tool of the RDP pyrosequencing pipeline http://pyro.cme.msu.edu/; [61]).

### Taxonomy assignment

In a first step, taxonomy was inferred for all reads using the stand-alone version of the RDP classifier (http://sourceforge.net/projects/rdp-classifier, revision 2.0, [62]). Taxon-level predictions were considered reliable when supported by a minimum bootstrap value of 80%. In order to predict taxonomy for each OTU, we either used any reference sequences present within a cluster, or the taxonomy of the reads present in the cluster, as predicted by the RDP classifier. To increase the resolution of the prediction, we privileged any reference sequences over the reads. For each OTU, taxonomy was inferred by a simple majority vote: if more than half of the reference sequences (or reads) present within a cluster agreed on a taxon, the OTU was annotated according to this taxon. In case of conflicts, we assigned a consensus taxon to a higher phylogenetic level for which the majority vote condition was met.

### Chimera estimation

Deep pyrosequencing on the 454 platform has revealed extensive microbial diversity that was previously undetected with culture-dependent methods [63]. Nevertheless, sequencing data generated from pools of PCR products have to be interpreted carefully; limitations and biases of the PCR technique have to be taken into account. This can lead to over-estimations of microbial diversity as has been recently reported [58], [64]. Moreover, during amplification, chimerical sequences can be generated.

On such short sequences, recombination points (recombination can occur from an incompletely extended primer or by template-switching; [65]) are extremely difficult to detect. Recently, a new tool to filter noise and remove chimera in 454 pyrosequencing data has been published [64]. There, the authors suggest that because of sequencing errors, diversity estimates may be at least an order of magnitude too high. To our best knowledge, at the time of our analysis, there were no available tools to detect chimera within libraries of short 454 reads. Therefore, in order to detect chimeras we decided to compare taxonomies assigned to N-terminal and C-terminal read fragments using BLASTn. In order to ensure a reasonable alignment length and a relatively high identity to the matching reference sequences, we only analyzed reads for which both fragments had a minimum identity of 95% and a minimum bit-score of 150 (these cutoffs were selected heuristically). A given read was deemed chimeric when the taxonomies of the best hits of each half were clearly not congruent (i.e., differing at the phylum level). Our simple chimera detection method resulted in a slightly higher rate of detected chimera compared to the method of Quince et *al.*, 2009 (∼4.5% compare to ∼3% in their example), suggesting that our approach is at least of comparable stringency [64].

## Supporting Information

Figure S1 ‘Bacterial FACS’ assay for detection of a *Salmonella*-surface-specific immunoglobulins (Ig). **A.** Experimental design for detection of bacteria specific antibodies via FACS analysis. Antibodies directed against the surface of *Salmonella typhimurium* in murine serum or intestinal lavage were detected by bacterial FACS (Materials and Methods for details) as described previously [30]. Overnight cultures of *S. tm* ^att^ (approx. 10^6^ cfu) were first incubated with decreasing dilutions (1∶180, 1∶60, 1∶20) of serum from a naïve (upper row) or a mouse at day 21 p.i. with *S. tm* ^att^ (lower row). Bacteria-bound IgG was then detected using an anti-mouse-IgG-PE antibody (or α-mouse-IgA-FITC, not shown). **Left panel:** Bacterial gate determined in FSC/SSC (forward-/sideward scatter). **Other panels:** Dot plot (FSC/FL2) showing IgG-PE^+^ bacteria. From these data, we calculated the *Salmonella*-specific Ig (relative fluorescence units) (Materials and Methods; Supplemental Fig1C and Fig.2C, Fig.6B): *S. tm*-specific Ig = (Mean fluorescence intensity of Ig^+^ bacteria**^§^**) * (% of positively stained bacteria of all bacteria^*^). The percentage of positively stained bacteria of all bacteria at the respective dilution is shown in each FACS plot. The mean fluorescence intensity of the Ig^+^ bacteria is shown below each FACS plot. **B.** Antibodies specifically detect *S. tm* but not *E. coli*. *S. tm* ^att^ or *E. coli* were incubated with increasing dilutions (1∶2; 1∶6; 1∶18) of an intestinal lavage obtained from a mouse at day 21 p.i. with *S. tm* ^att^. Bacteria-bound IgA was detected by anti-mouse-IgA-FITC. **Upper panel:** Dot plot (FSC/FL1) showing IgA-FITC^+^ *S. tm* ^att^. **Lower panel:** Dot plot (FSC/FL1) showing IgA-FITC^+^ *E.coli*. **C.** Plotting *Salmonella*-specific antibodies. *S*pecific Ig (rfu) against *S. tm^att^* (*S. tm^att^* coated; experiment) or *E. coli* (*E. coli* coated; neg. control) for decreasing dilutions (indicated as black slope) of serum (1∶180, 1∶60, 1∶20) or gut wash (1∶18, 1∶6, 1∶2) at day 21 post *S. tm^att^* immunization were calculated as described in Supplemental Fig.1A and plotted for indicated mice.
(0.20 MB TIF)

Figure S2 Immunohistological analysis of *S. tm* ^att^-immunized mice is in line with an adaptive mucosal immune response. **A**. Immunohistological analysis of naïve C57BL/6 mice (conventional SPF gut flora; C-mice) and of mice at day 40 p.i. with *S. tm* ^att^. Quantitative data of Ly6G^+^, IgA^+^, CD4^+^ and CD8^+^ cells in cecal tissue of naïve C57BL/6 and day 40 *S. tm* ^att^-immunized mice were obtained by counting 15 randomly selected 40× high power fields (hpf) from 3 mice per group. Y-axis: average cell number per 40× hpf. **B**. Immunohistochemical stainings for Ly6G, IgA, CD4 and CD8 markers were prepared as indicated in Materials and Methods. Scale bar = 50µm.
(6.86 MB TIF)

Figure S3 *S. tm* ^att^ immunized mice mount an O-antigen specific IgA response. Serum from the same naïve and *S. tm* ^att^ infected mice (day 40 p.i.) as shown in Fig. 2D was analyzed by immunoblot against different bacterial lysates (*S. tm* ^ΔO^; *L. reuteri*; *E. faecalis*; *S. en* ^wt^; *E. coli*; *S. tm* ^att^; *S. tm* ^att^ digested with proteinase K; *S. tm* M933 [no flagella, no functional TTSS]; flagellin FliC). IgA was detected with a goat-anti-mouse-IgA-HRP secondary antibody. The experiment is representative for 6 different animals.
(0.68 MB TIF)

Figure S4 Serum of a *S. tm* infected human patient (‘asymptomatic excretor’) shows a *S. tm*-specific serum Ig-response. The human patient had *S. typhimurium*-positive stool cultures for at least two months. The specificity of the Ig-response in the serum was tested by immunoblot against different bacterial lysates (*S. tm* ^ΔO^; *L. reuteri*; *E. faecalis*; *S. en* ^wt^; *E. coli*; *S. tm* ^att^; *S. tm* ^att^ digested with proteinase K; *S. tm* M933 [no flagella, no functional TTSS]; flagellin FliC). Bound human Igs were detected using anti-human-IgA-HRP and anti-human-IgG-HRP secondary antibodies. The patient serum revealed *S. tm*- (but not *S. en*)-O-antigen-specific IgA- and IgG-responses. We speculate that the *E. coli*-O-antigen-specific antibodies might be attributable to a previous exposure to pathogenic *E. coli* spp.. However, this has not been analyzed.
(1.00 MB TIF)

Figure S5 An acute inflammatory response in the gut might be required for the induction of *S. tm*-specific mucosal IgA. Non-sm-treated *S. tm* ^att^ immunized, sm-treated *S. tm* ^avir^-immunized as well as *S. tm* ^att^ i.v. immunized mice do not mount a *Salmonella*-specific adaptive IgA response and are not protected against colitis upon challenge with *S. tm* ^wt^. **A**. The first group of C57BL/6 mice (n = 5; black symbols) was pretreated with sm and immunized with *S. tm* ^avir^ (5×10^7^ cfu by gavage). The second group of C57BL/6 mice (n = 5; blue symbols) was not pretreated with sm and immunized with *S. tm* ^att^ (5×10^7^ cfu by gavage). The third group of C57BL/6 mice (n = 5; green symbols) was not pretreated with sm and immunized with *S. tm* ^att^ (5×10^5^ cfu i.v.). At day 40 p.i. mice were treated with ampicillin and orally challenged with *S. tm* ^wt^ (200 cfu by gavage). Mice were sacrificed at day 2 post challenge and *Salmonella* loads in the cecal content (left panel) and the MLN (middle panel) were determined. Cecal pathology was evaluated (right panel). **B**. *Salmonella*-specific sIgA response. Ig-specific antibody responses against different bacterial lysates (*S. tm* ^ΔO^; *L. reuteri*; *E. faecalis*; *S. en* ^wt^; *E. coli*; *S. tm* ^att^; *S. tm* ^att^ digested with proteinase K; *S. tm* M933 [no flagella, no functional TTSS]; flagellin FliC) were tested by immunoblot analysis. Immunoblots were incubated with serum or gut wash of sm-treated *S. tm* ^avir^ immunized mice, non-sm-treated *S. tm* ^att^-immunized or *S. tm* ^att^ i.v. immunized mice that were orally challenged with *S. tm* ^wt^ at day 40 p.i.. Specific antibodies were detected with an anti-mouse-IgA-HRP conjugate.
(1.27 MB TIF)

Figure S6 Antibody responses of *S. tm* ^att^ immunized J_H_^−/−^ IgA^−/−^ , pIgR^−/−^ and TCRβ^−/−^δ^−/−^ mice. The specific Ig-response of the mice shown in Table 1 (day 40 post immunization with *S. tm* ^att^) was analyzed by Western blot using different bacterial lysates (*S. tm* ^ΔO^; *L. reuteri*; *E. faecalis*; *S. en* ^wt^; *E. coli*; *S. tm* ^att^; *S. tm* ^att^ digested with proteinase K; *S. tm* M933 [no flagella, no functional TTSS]; flagellin FliC). Serum or gut wash of d 40 *S. tm* ^att^-immunized knockout mice was tested and specific antibodies were detected with anti-mouse-IgA-HRP or anti-mouse-IgG-HRP conjugates. Panels show specificity of Ig in serum and gut wash of the indicated knock-out mice. Slight amounts of *S. tm* specific sIgA were detected in the gut wash of pIgR^−/−^ mice. We speculate that this is attributable to the 10-fold increased serum IgA levels and consequent leakage in to the gut lumen as described before [18], [66] and our own data (not shown).
(3.32 MB TIF)

Figure S7 Serovar specificity of the anti-LPS antibody response. *S. tm* ^att^ and *S. en* ^att^ immunized C57BL/6 mice mount a serovar-specific serum IgA response by day 40 p.i.. Sera of *Salmonella*-or mock-immunized mice were analyzed by immunoblot using anti-mouse-IgA-HRP antibody against lysates of *S. tm* ^att^ and *S.en*, respectively. **Left panel:** Serum of a *S. tm* ^att^-immunized C57BL/6 mouse. **Middle panel:** Serum of a *S. en* ^att^-immunized C57BL/6 mouse. **Right panel:** Serum of a mock immunized C57BL/6 mouse.
(0.32 MB TIF)

Figure S8 L-mice mount a LPS-O-antigen-specific sIgA response by day 40 post *S. tm* ^att^ infection. The specific Ig-response of the mice shown in Fig. 4A (day 40 post immunization with *S. tm* ^att^) was analyzed by Western blot using different bacterial lysates (*S. tm* ^ΔO^; *L. reuteri*; *E. faecalis*; *S. en* ^wt^; *E. coli*; *S. tm* ^att^; *S. tm* ^att^ digested with proteinase K; *S. tm* M933 [no flagella, no functional TTSS]; flagellin FliC). Serum or gut wash was tested and specific antibodies were detected with anti-mouse-IgA-HRP or anti-mouse-IgG-HRP conjugates.
(0.58 MB TIF)

Figure S9 Protein association network of genes significantly upregulated in the cecal mucosa of *S. tm* ^att^→L mice. Gene expression profiles of the cecal mucosa of naïve L and *S. tm* ^att^→L mice (day 40) were determined using mouse gene expression microarrays (see Materials and Methods; Supplemental Table 3). Genes significantly up-regulated in the cecal mucosa of *S. tm* ^att^→L mice at day 40 p.i. (compared to naïve L-mice) were determined (significance of log_2_ fold changes *p*<0.001) and protein functional interactions visualized using STRING version 8.2 [67]. Colors denote the results of unsupervised clustering of the interaction network, the resulting clusters are indicative of proteins with related biological function and are annotated by GO categories assigned to genes detected as significantly up-regulated. For some gene groups, biological functions were added to ease understanding (i.e. T-cell-mediated immunity, immunity and defense, MHCI, MHCII, B-cell and antibody mediated immunity, cytokines and chemokines). Parameters used in STRING (Active Prediction Methods (connecting lines): Text mining (yellow), Neighborhood (green), Gene Fusion (red), Co-occurrence (blue), Co-expression (black), Experiments (purple), Databases (turquoise) ; confidence score: 0.15; Network clustering (KMeans: 10).
(0.42 MB TIF)

Table S1 Bacterial strains and plasmids used in this study.
(0.36 MB PDF)

Table S2 Mice used in this study.
(0.19 MB PDF)

Table S3 Pathways significantly over-represented among the differentially expressed genes in the cecal mucosa of L-mice at day 40 p.i. with *S. tm* ^att^.
(0.47 MB PDF)

Table S4 Quantitative microbiota analysis by 454 amplicon sequencing.
(0.39 MB PDF)
