# Supplementary figures and images for: The Microbiota Mediates Pathogen Clearance from the Gut Lumen after Non-Typhoidal Salmonella Diarrhea
Source: PLoS Pathog. 2010 Sep 9;6(9):e1001097. doi: 10.1371/journal.ppat.1001097 (PMC2936549; doi:10.1371/journal.ppat.1001097)

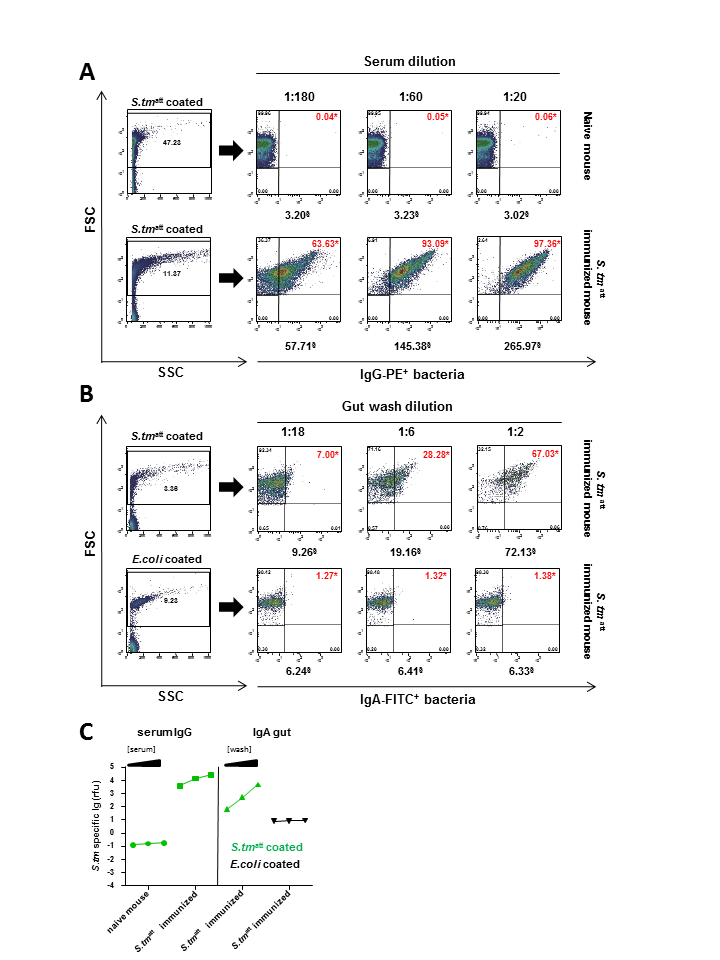

Supplement: Figure S1 — ‘Bacterial FACS’ assay for detection of a Salmonella-surface-specific immunoglobulins (Ig). A. Experimental design for detection of bacteria specific antibodies via FACS analysis. Antibodies directed against the surface of Salmonella typhimurium in murine serum or intestinal lavage were detected by bacterial FACS (Materials and Methods for details) as described previously [30]. Overnight cultures of S. tm att (approx. 106 cfu) were first incubated with decreasing dilutions (1∶180, 1∶60, 1∶20) of serum from a naïve (upper row) or a mouse at day 21 p.i. with S. tm att (lower row). Bacteria-bound IgG was then detected using an anti-mouse-IgG-PE antibody (or α-mouse-IgA-FITC, not shown). Left panel: Bacterial gate determined in FSC/SSC (forward-/sideward scatter). Other panels: Dot plot (FSC/FL2) showing IgG-PE+ bacteria. From these data, we calculated the Salmonella-specific Ig (relative fluorescence units) (Materials and Methods; Supplemental Fig1C and Fig.2C, Fig.6B): S. tm-specific Ig = (Mean fluorescence intensity of Ig+ bacteria§) * (% of positively stained bacteria of all bacteria*). The percentage of positively stained bacteria of all bacteria at the respective dilution is shown in each FACS plot. The mean fluorescence intensity of the Ig+ bacteria is shown below each FACS plot. B. Antibodies specifically detect S. tm but not E. coli. S. tm att or E. coli were incubated with increasing dilutions (1∶2; 1∶6; 1∶18) of an intestinal lavage obtained from a mouse at day 21 p.i. with S. tm att. Bacteria-bound IgA was detected by anti-mouse-IgA-FITC. Upper panel: Dot plot (FSC/FL1) showing IgA-FITC+ S. tm att. Lower panel: Dot plot (FSC/FL1) showing IgA-FITC+ E.coli. C. Plotting Salmonella-specific antibodies. Specific Ig (rfu) against S. tmatt (S. tmatt coated; experiment) or E. coli (E. coli coated; neg. control) for decreasing dilutions (indicated as black slope) of serum (1∶180, 1∶60, 1∶20) or gut wash (1∶18, 1∶6, 1∶2) at day 21 post S. tmatt immunization were calcu [file ppat.1001097.s001.tif]

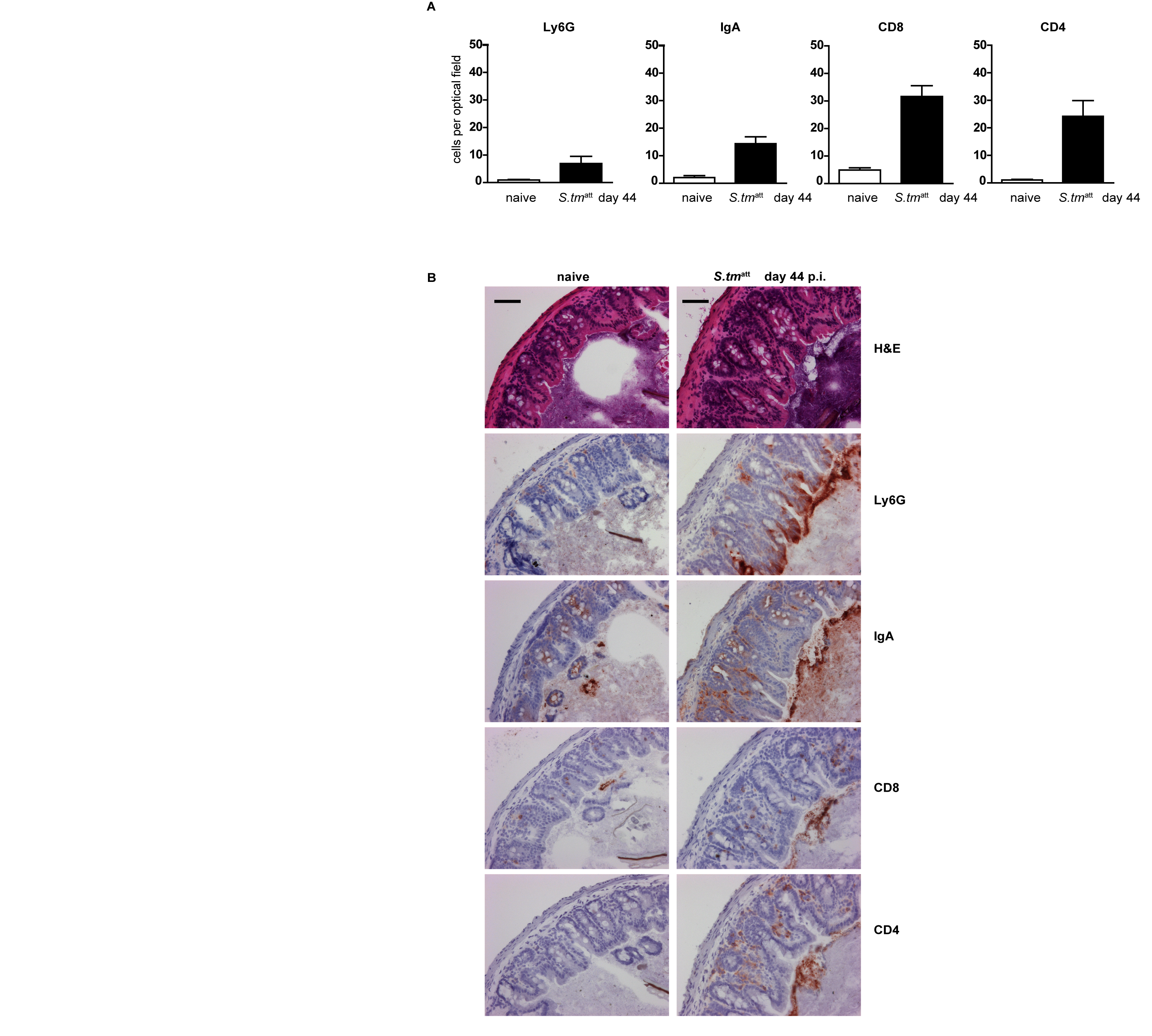

Supplement: Figure S2 — Immunohistological analysis of S. tm att-immunized mice is in line with an adaptive mucosal immune response. A. Immunohistological analysis of naïve C57BL/6 mice (conventional SPF gut flora; C-mice) and of mice at day 40 p.i. with S. tm att. Quantitative data of Ly6G+, IgA+, CD4+ and CD8+ cells in cecal tissue of naïve C57BL/6 and day 40 S. tm att-immunized mice were obtained by counting 15 randomly selected 40× high power fields (hpf) from 3 mice per group. Y-axis: average cell number per 40× hpf. B. Immunohistochemical stainings for Ly6G, IgA, CD4 and CD8 markers were prepared as indicated in Materials and Methods. Scale bar = 50µm. (6.86 MB TIF) [file ppat.1001097.s002.tif]

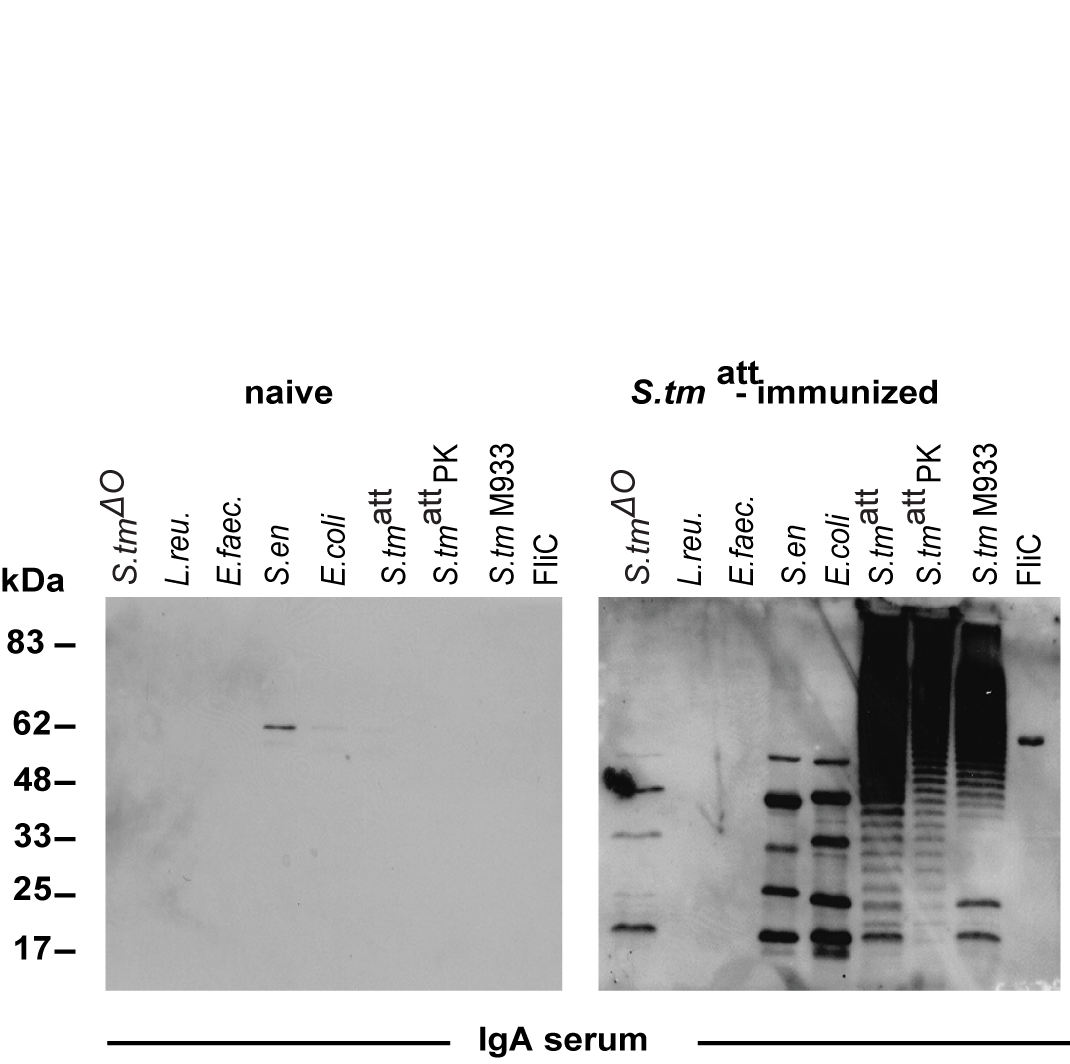

Supplement: Figure S3 — S. tm att immunized mice mount an O-antigen specific IgA response. Serum from the same naïve and S. tm att infected mice (day 40 p.i.) as shown in Fig. 2D was analyzed by immunoblot against different bacterial lysates (S. tm ΔO; L. reuteri; E. faecalis; S. en wt; E. coli; S. tm att; S. tm att digested with proteinase K; S. tm M933 [no flagella, no functional TTSS]; flagellin FliC). IgA was detected with a goat-anti-mouse-IgA-HRP secondary antibody. The experiment is representative for 6 different animals. (0.68 MB TIF) [file ppat.1001097.s003.tif]

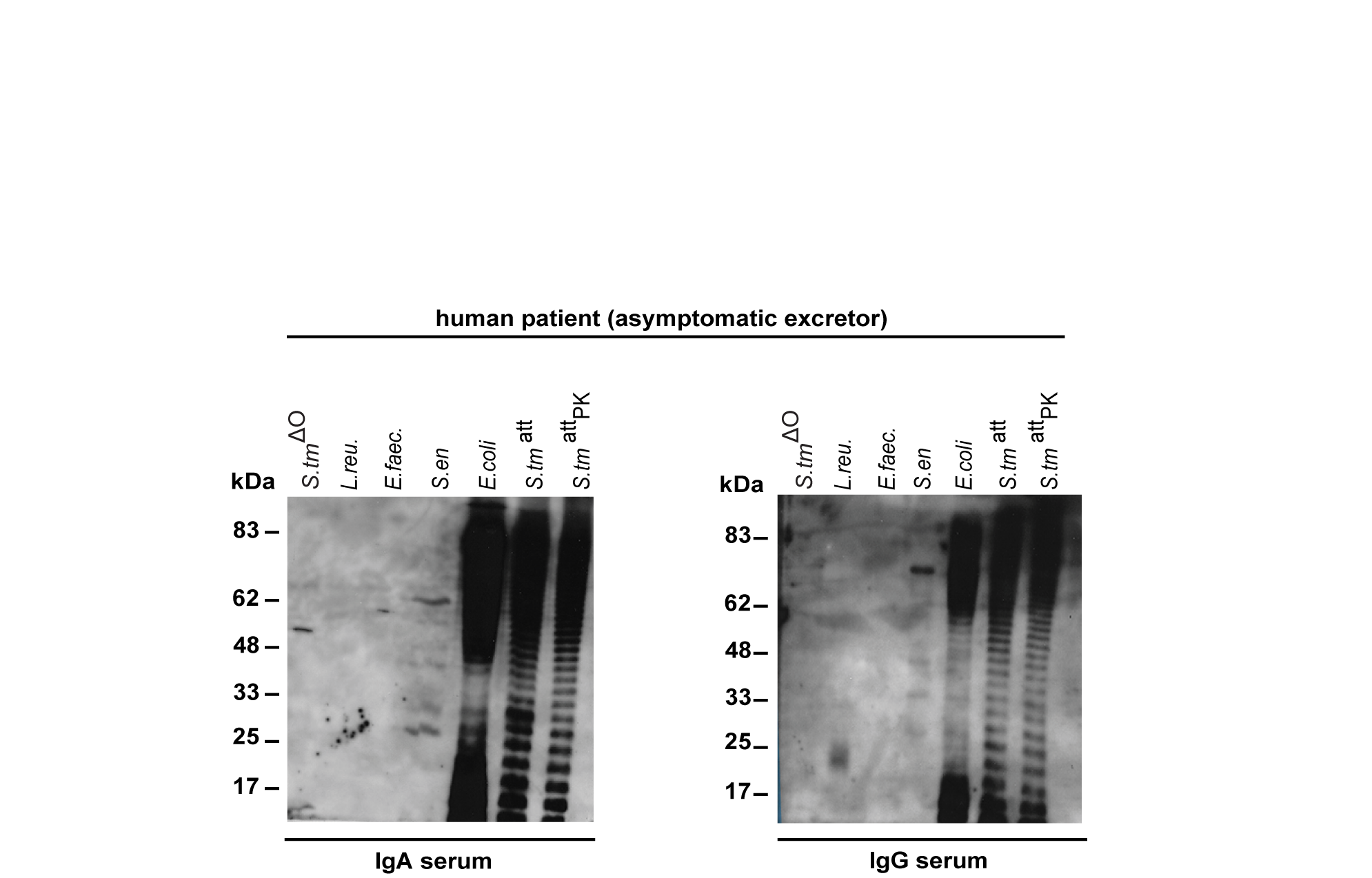

Supplement: Figure S4 — Serum of a S. tm infected human patient (‘asymptomatic excretor’) shows a S. tm-specific serum Ig-response. The human patient had S. typhimurium-positive stool cultures for at least two months. The specificity of the Ig-response in the serum was tested by immunoblot against different bacterial lysates (S. tm ΔO; L. reuteri; E. faecalis; S. en wt; E. coli; S. tm att; S. tm att digested with proteinase K; S. tm M933 [no flagella, no functional TTSS]; flagellin FliC). Bound human Igs were detected using anti-human-IgA-HRP and anti-human-IgG-HRP secondary antibodies. The patient serum revealed S. tm- (but not S. en)-O-antigen-specific IgA- and IgG-responses. We speculate that the E. coli-O-antigen-specific antibodies might be attributable to a previous exposure to pathogenic E. coli spp.. However, this has not been analyzed. (1.00 MB TIF) [file ppat.1001097.s004.tif]

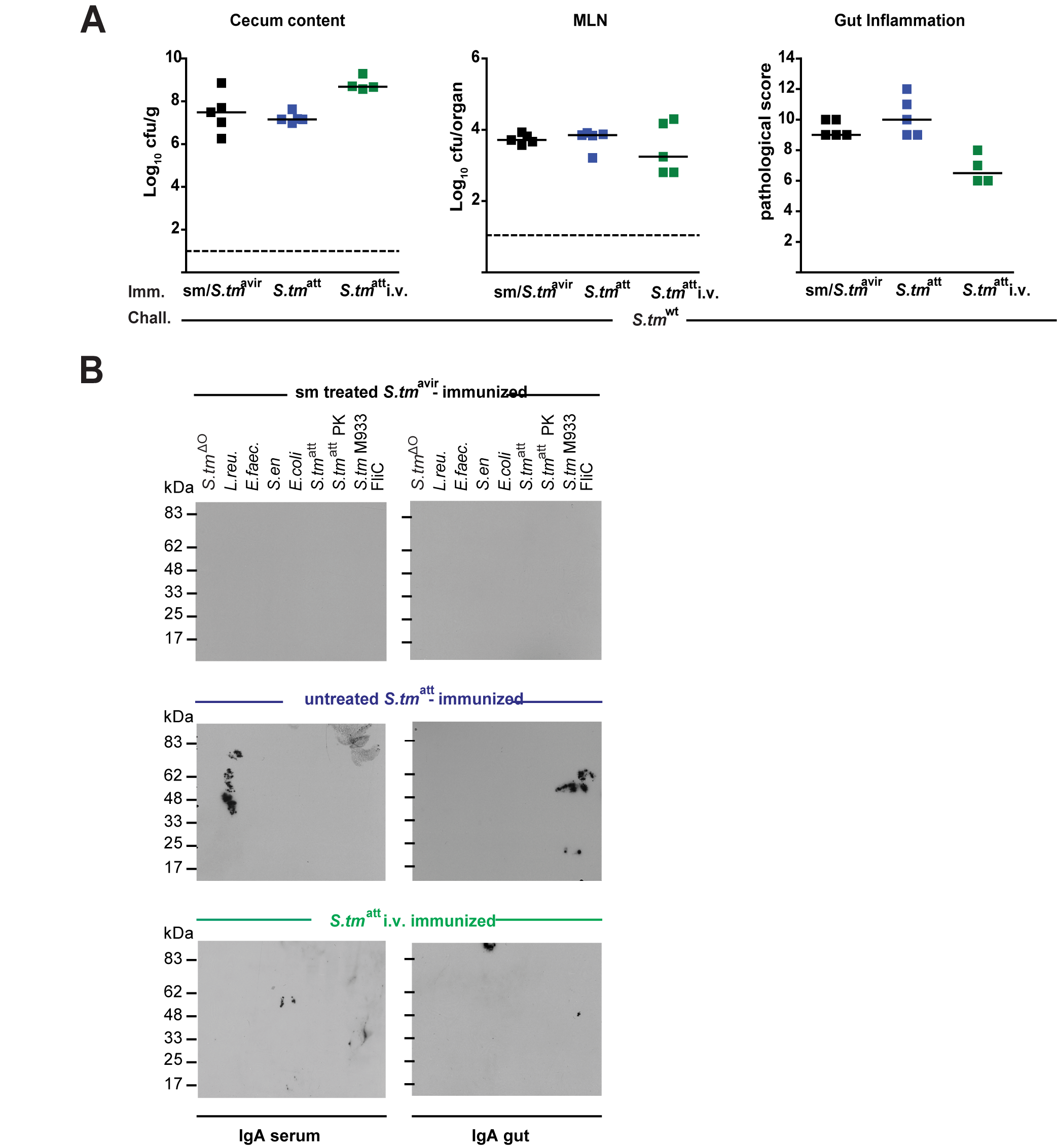

Supplement: Figure S5 — An acute inflammatory response in the gut might be required for the induction of S. tm-specific mucosal IgA. Non-sm-treated S. tm att immunized, sm-treated S. tm avir-immunized as well as S. tm att i.v. immunized mice do not mount a Salmonella-specific adaptive IgA response and are not protected against colitis upon challenge with S. tm wt. A. The first group of C57BL/6 mice (n = 5; black symbols) was pretreated with sm and immunized with S. tm avir (5×10^7^ cfu by gavage). The second group of C57BL/6 mice (n = 5; blue symbols) was not pretreated with sm and immunized with S. tm att (5×10^7^ cfu by gavage). The third group of C57BL/6 mice (n = 5; green symbols) was not pretreated with sm and immunized with S. tm att (5×10^5^ cfu i.v.). At day 40 p.i. mice were treated with ampicillin and orally challenged with S. tm wt (200 cfu by gavage). Mice were sacrificed at day 2 post challenge and Salmonella loads in the cecal content (left panel) and the MLN (middle panel) were determined. Cecal pathology was evaluated (right panel). B. Salmonella-specific sIgA response. Ig-specific antibody responses against different bacterial lysates (S. tm ΔO; L. reuteri; E. faecalis; S. en wt; E. coli; S. tm att; S. tm att digested with proteinase K; S. tm M933 [no flagella, no functional TTSS]; flagellin FliC) were tested by immunoblot analysis. Immunoblots were incubated with serum or gut wash of sm-treated S. tm avir immunized mice, non-sm-treated S. tm att-immunized or S. tm att i.v. immunized mice that were orally challenged with S. tm wt at day 40 p.i.. Specific antibodies were detected with an anti-mouse-IgA-HRP conjugate. (1.27 MB TIF) [file ppat.1001097.s005.tif]

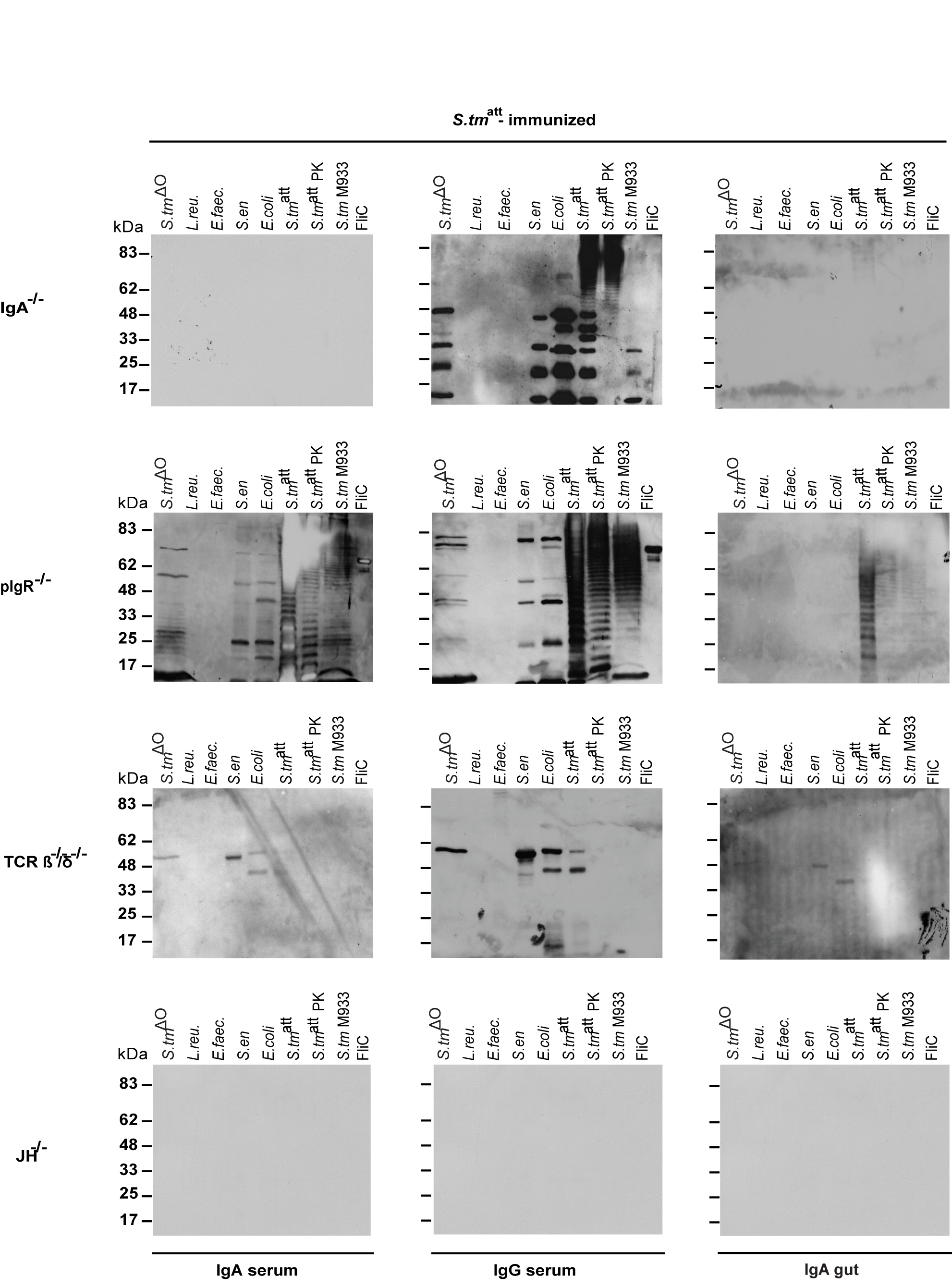

Supplement: Figure S6 — Antibody responses of S. tm att immunized JH −/− IgA−/− , pIgR−/− and TCRβ−/−δ−/− mice. The specific Ig-response of the mice shown in Table 1 (day 40 post immunization with S. tm att) was analyzed by Western blot using different bacterial lysates (S. tm ΔO; L. reuteri; E. faecalis; S. en wt; E. coli; S. tm att; S. tm att digested with proteinase K; S. tm M933 [no flagella, no functional TTSS]; flagellin FliC). Serum or gut wash of d 40 S. tm att-immunized knockout mice was tested and specific antibodies were detected with anti-mouse-IgA-HRP or anti-mouse-IgG-HRP conjugates. Panels show specificity of Ig in serum and gut wash of the indicated knock-out mice. Slight amounts of S. tm specific sIgA were detected in the gut wash of pIgR−/− mice. We speculate that this is attributable to the 10-fold increased serum IgA levels and consequent leakage in to the gut lumen as described before [18], [66] and our own data (not shown). (3.32 MB TIF) [file ppat.1001097.s006.tif]

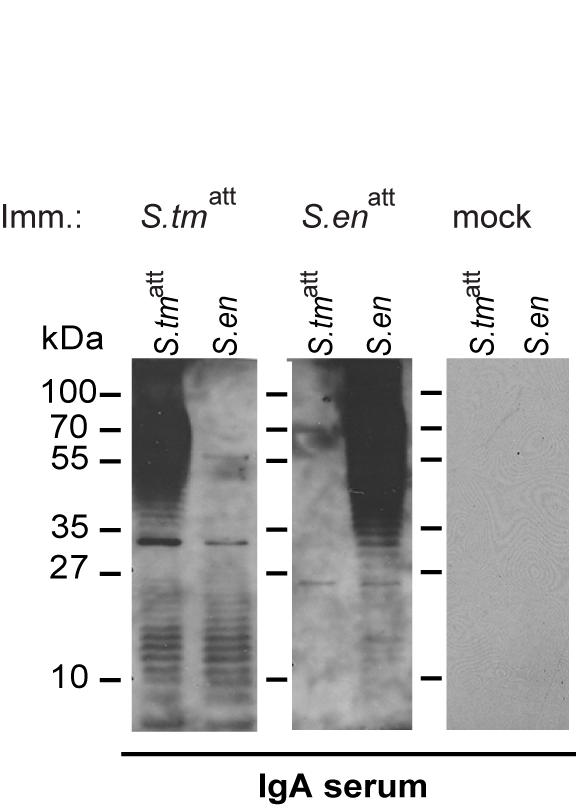

Supplement: Figure S7 — Serovar specificity of the anti-LPS antibody response. S. tm att and S. en att immunized C57BL/6 mice mount a serovar-specific serum IgA response by day 40 p.i.. Sera of Salmonella-or mock-immunized mice were analyzed by immunoblot using anti-mouse-IgA-HRP antibody against lysates of S. tm att and S.en, respectively. Left panel: Serum of a S. tm att-immunized C57BL/6 mouse. Middle panel: Serum of a S. en att-immunized C57BL/6 mouse. Right panel: Serum of a mock immunized C57BL/6 mouse. (0.32 MB TIF) [file ppat.1001097.s007.tif]

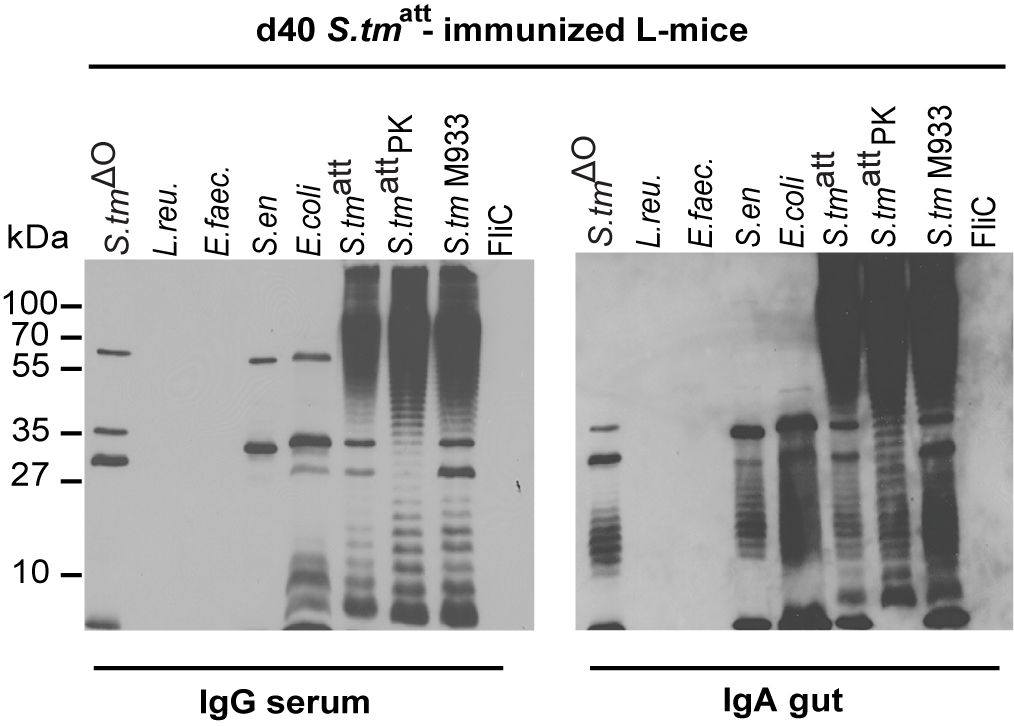

Supplement: Figure S8 — L-mice mount a LPS-O-antigen-specific sIgA response by day 40 post S. tm att infection. The specific Ig-response of the mice shown in Fig. 4A (day 40 post immunization with S. tm att) was analyzed by Western blot using different bacterial lysates (S. tm ΔO; L. reuteri; E. faecalis; S. en wt; E. coli; S. tm att; S. tm att digested with proteinase K; S. tm M933 [no flagella, no functional TTSS]; flagellin FliC). Serum or gut wash was tested and specific antibodies were detected with anti-mouse-IgA-HRP or anti-mouse-IgG-HRP conjugates. (0.58 MB TIF) [file ppat.1001097.s008.tif]

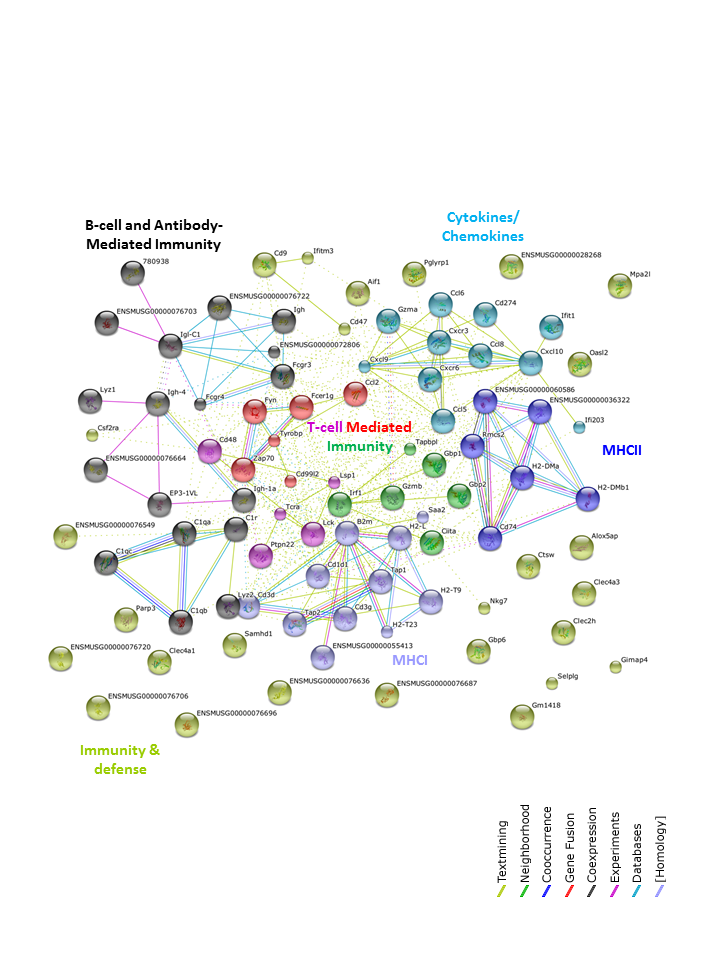

Supplement: Figure S9 — Protein association network of genes significantly upregulated in the cecal mucosa of S. tm att→L mice. Gene expression profiles of the cecal mucosa of naïve L and S. tm att→L mice (day 40) were determined using mouse gene expression microarrays (see Materials and Methods; Supplemental Table 3). Genes significantly up-regulated in the cecal mucosa of S. tm att→L mice at day 40 p.i. (compared to naïve L-mice) were determined (significance of log2 fold changes p<0.001) and protein functional interactions visualized using STRING version 8.2 [67]. Colors denote the results of unsupervised clustering of the interaction network, the resulting clusters are indicative of proteins with related biological function and are annotated by GO categories assigned to genes detected as significantly up-regulated. For some gene groups, biological functions were added to ease understanding (i.e. T-cell-mediated immunity, immunity and defense, MHCI, MHCII, B-cell and antibody mediated immunity, cytokines and chemokines). Parameters used in STRING (Active Prediction Methods (connecting lines): Text mining (yellow), Neighborhood (green), Gene Fusion (red), Co-occurrence (blue), Co-expression (black), Experiments (purple), Databases (turquoise) ; confidence score: 0.15; Network clustering (KMeans: 10). (0.42 MB TIF) [file ppat.1001097.s009.tif]
