# Supplementary material for: The Microbiota Mediates Pathogen Clearance from the Gut Lumen after Non-Typhoidal Salmonella Diarrhea
Source: PLoS Pathog. 2010 Sep 9;6(9):e1001097. doi: 10.1371/journal.ppat.1001097 (PMC2936549; doi:10.1371/journal.ppat.1001097)
Supplement: Table S1 — Bacterial strains and plasmids used in this study. (0.36 MB PDF) [file ppat.1001097.s010.pdf]

Supplemental Figures

The microbiota mediates pathogen clearance from the gut lumen after non-typhoidal *Salmonella* diarrhea

Kathrin Endt*[1], Bärbel Stecher*[1], Samuel Chaffron[2], Emma Slack[3], Nicolas Tchitchek[4], Arndt Benecke[4], Laurye Van Maele[5], Jean-Claude Sirard[5], Andreas J. Mueller[1], Mathias Heikenwalder[6], Andrew J. Macpherson[3], Richard Strugnell[7], Christian von Mering[2] and Wolf-Dietrich Hardt[1#]

Table S1. **Bacterial strains and Plasmids used in this study**

Strain	Genotype and antibiotic resistance	Phenotype	Source or reference
S. tm ^{wt}	*S. tm* strain SL1344; strep^{R}	*S.typhimurium* wild type	[1]
S. tm^{att} (M556)	SL1344 *sseD*::*aphT;* strep^{R}, km^{R}	Induces gut inflammation via the SPI-1 TTSS; Lacks SPI-2 TTSS; attenuated in systemic infection	[2]
S. tm $^{\Delta O}$	SL1344Δ*wbaP;* strep^{R}	No O-antigen	[3]
S. tm^{avir} (M557)	SL1344 Δ*invG* *sseD::aphT,* strep^{R}, km^{R}	Lacks functional SPI-1 and SPI-2 TTSS; does not induce gut inflammation and is attenuated in systemic infection	[2]
M933	SL1344 Δ*invG*; *sseD*::*aphT*; *fliGHI*::Tn*10*; strep^{R}, km^{R}, tet^{R}	Lacks functional SPI-1, SPI-2 TTSS and flagella biosynthesis (no flagellin produced)	[4]
S.en wt	*S.en* strain 125109; strep^{R}	*S.enteritidis* wild type	[5]
S.en^{att} (M1512)	*S.en* 125109 Δ*ssaV::cat;* strep^{R}, cm^{R}	Induces gut inflammation via the SPI-1 TTSS; Lacks SPI-2 TTSS; attenuated in systemic infection	[5]
S.en^{avir} (M1513)	*S.en* 125109 *invC*::aphT; *ssaV::cat;* strep^{R}, km^{R}, cm^{R}	Lacks functional SPI-1 and SPI-2 TTSS; does not induce gut inflammation and is attenuated in systemic infection	[5]
Escherichia coli	*Nissle 1917 strain; wild type*		Kind gift of Dr. Sören

			Schubert
Lactobacillus reuteri	*Wild type*	Mouse gut isolate	[6]
Enterococcus faecalis	*Wild type*	Mouse gut isolate	This study
**Plasmid**	**Genotype and antibiotic resistance**	**Phenotype**	**Source or reference**
pM979	P_{rpsM} *gfpmut2* *ori*pBR322; amp^R	Constitutive *gfp* expression	[7]
pM973	P_{ssaG} *gfpmut2* *ori*pMB1 amp^R	Intracellular *gfp* expression; Indicator for Salmonella invaded into the gut mucosa; Used for 'challenge infections'	[8]
pWKS30	amp^R; *ori*pMB1		[9]
