# Supplementary material for: The Microbiota Mediates Pathogen Clearance from the Gut Lumen after Non-Typhoidal Salmonella Diarrhea
Source: PLoS Pathog. 2010 Sep 9;6(9):e1001097. doi: 10.1371/journal.ppat.1001097 (PMC2936549; doi:10.1371/journal.ppat.1001097)
Supplement: Table S2 — Mice used in this study. (0.19 MB PDF) [file ppat.1001097.s011.pdf]

Table S2. Mice used in this study

Name	genetic background	microbiota*	colonization resistance	source or reference
Wild type mice (C)	C57BL/6	specific pathogen free (C)	yes	Rodent center HCI (RCHCI)
TCR $\beta^{-/-}\delta^{-/-}$	C57BL/6	specific pathogen free (C)	yes	[10]
JHT	C57BL/6	specific pathogen free (C)	yes	[11]
$IgA^{-/-}$	C57BL/6	specific pathogen free (C)	yes	[12]
$pIgR^{-/-}$	C57BL/6	specific pathogen free (C)	yes	[13]
L (LCM)	C57BL/6	low complexity microbiota	no	[14]

*C= conventional, specified pathogen free microbiota.
