# Supplementary material for: The Microbiota Mediates Pathogen Clearance from the Gut Lumen after Non-Typhoidal Salmonella Diarrhea
Source: PLoS Pathog. 2010 Sep 9;6(9):e1001097. doi: 10.1371/journal.ppat.1001097 (PMC2936549; doi:10.1371/journal.ppat.1001097)
Supplement: Table S3 — Pathways significantly over-represented among the differentially expressed genes in the cecal mucosa of L-mice at day 40 p.i. with S. tm att. (0.47 MB PDF) [file ppat.1001097.s012.pdf]

Table S3. Pathways significantly over-represented among the differentially expressed genes in the cecal mucosa of L-mice at day 40 p.i. with *S. tm* ^{att}

B-cell- and antibody-mediated immunity

PROBE	LOG_Q[090514_J-K_ind.ma2]	VAR[090514_J-K_ind.ma2]	P[090514_J-K_ind.ma2]	Celera Gene ID	Gene_Symbol	Gene_Name
429239	5.93180	0.93180	0.00000	mCG56372.2	LOC432699	null
339582	4.26380	0.89670	0.00000	mCG140410	Igh-1a|LOC544908	immunoglobulin heavy chain 1a (serum IgG2a)
590082	4.13340	0.85190	0.00000	mCG141789.1	Igk-V32	immunoglobulin kappa chain variable 32 (V32)
808065	3.99290	1.80190	0.00000	mCG130744.2|mCG1036427.2	LOC635601|bd2|LOC628027	null
736226	3.97930	1.02240	0.00000	mCG1027522.1|mCG1029835.1	LOC636462	null
401576	3.90870	1.76620	0.00000	mCG132002.2	LOC243439	null
891891	3.85240	1.34100	0.00000	mCG113660.1	LOC633515	null
538665	3.83340	0.58660	0.00000	null	LOC434031|Igk-V38	immunoglobulin kappa chain variable 38(V38)
491036	3.77740	0.84550	0.00000	mCG116913.1	LOC636067	null
699266	3.77400	0.76790	0.00000	mCG1025451.1	null	null
742698	3.74840	0.88410	0.00000	mCG130744.2|mCG1036427.2|mCG1036498.2	LOC635664|LOC635601|bd2|LOC628056|LOC628027	null
493584	3.71590	0.98470	0.00000	mCG142153.1	LOC620105|LOC637047	null
411187	3.70140	0.56530	0.00000	null	LOC620017	null
348117	3.67230	0.45770	0.00000	mCG1036432.2	null	null
857940	3.65340	1.28650	0.00000	mCG1037662.1	LOC630251	null
715331	3.64830	0.51560	0.00000	mCG141621.1	LOC434037	null
901486	3.64130	1.45140	0.00000	mCG68149.2	LOC630331|LOC633472	null
596021	3.63320	1.11870	0.00000	mCG146921.1|mCG141794.1|mCG141802.1	IgM	null
574582	3.58730	1.10810	0.00000	mCG146921.1|mCG141794.1	IgM	null
515370	3.58010	1.03810	0.00000	mCG142162.1	LOC626145|LOC545854	null
444829	3.55200	1.23660	0.00000	mCG141790.1	Gm1067	gene model 1067, (NCBI)
489583	3.43750	0.72290	0.00000	mCG114300.1	null	null
479671	3.42090	0.74830	0.00000	mCG126269.2|mCG1036505.2	gn33|Igk-V34|Igk-V33	immunoglobulin kappa chain variable 34 (V34)|immunoglobulin kappa chain variable 33 (V33)
896758	3.41680	0.08000	0.00000	mCG1037964.1	LOC620216|LOC632220	null
583890	3.36590	0.47530	0.00000	mCG117763.1	LOC634652|LOC382695|LOC436124	null
919480	3.30590	0.97960	0.00000	mCG141784.1	LOC434025	null
675426	3.26900	0.99890	0.00000	mCG128543.1	Igh-1a|LOC382692|LOC628614	immunoglobulin heavy chain 1a (serum IgG2a)
823783	3.23050	0.56160	0.00000	mCG1036439.2	LOC384413|Igk-V28	immunoglobulin kappa chain variable 28 (V28)
899105	3.22980	0.41100	0.00000	mCG142070	LOC637785	null
739642	3.18500	0.52560	0.00000	mCG141793.1	LOC434026	null
898268	3.16270	0.52810	0.00000	mCG127559.1|mCG114359	LOC640614|LOC632997|LOC636453	null
317586	3.15470	0.98640	0.00010	mCG1025125.1	LOC631518	null
576556	3.14730	1.11630	0.00000	mCG140401	LOC634430	null
902557	3.11220	0.71020	0.00000	mCG127559.1	null	null
843602	3.08470	0.78140	0.00000	mCG118954	LOC638634	null
897561	3.08440	0.54880	0.00000	null	LOC434609	null
483305	3.08070	0.65440	0.00000	null	Gm1419	gene model 1419, (NCBI)
901720	3.07910	0.51480	0.00000	mCG1026782.1	LOC382695|LOC629938|LOC638485	null
523804	3.01550	0.22180	0.00000	mCG1037963.1	LOC632180|LOC432993|LOC620169	null
875823	2.97880	0.64400	0.00000	mCG129378	LOC544903	null
410888	2.95830	0.97940	0.00000	mCG1025140.1	LOC633734	null
672387	2.94750	1.17050	0.00000	mCG114360|mCG1025864.1	null	null
401803	2.93560	0.50790	0.00000	mCG118868.1	LOC634298	null
615665	2.92850	0.88010	0.00000	mCG1050179	D6Mit97|Igk-V28	DNA segment, Chr 6, Massachusetts Institute of Technology 97|immunoglobulin kappa chain variable 28 (V28)
579252	2.92670	0.63600	0.00000	mCG127631.1	Igh-4	immunoglobulin heavy chain 4 (serum IgG1)
832392	2.90250	0.86180	0.00000	null	LOC628127	null
894906	2.89830	0.71310	0.00000	mCG118865.1|mCG114299.1	LOC629884|LOC637000|Igh|Igh-VJ558	immunoglobulin heavy chain complex|immunoglobulin heavy chain (J558 family)
899101	2.79970	1.08000	0.00000	mCG129262.1	Igh-VJ558	immunoglobulin heavy chain (J558 family)
893766	2.79370	0.47810	0.00000	mCG116911.1|mCG142745|mCG142069	LOC619833|LOC382696|LOC637398|LOC637794|VH18	immunoglobulin heavy chain (J558 family)
894845	2.79170	0.66090	0.00000	mCG1036509.2|mCG141633.1|mCG141637.1	LOC628516|LOC636646|LOC636598|LOC628498	null
621943	2.75070	0.43730	0.00000	mCG127630.1	LOC633062	null
797351	2.73710	0.26570	0.00000	mCG118871.1|mCG114299.1|mCG140407	Igh-1a|LOC634338|LOC629884|LOC629871|LOC63422	immunoglobulin heavy chain 1a (serum IgG2a)|immunoglobulin heavy chain complex|immunoglobulin heavy chain (J558 family)
721976	2.72350	0.78620	0.00000	mCG141796.1	LOC545847	null
892758	2.68780	0.73590	0.00000	mCG118870.1	LOC634541	null
685068	2.68190	0.96900	0.00000	mCG131876.2	null	null
902558	2.67750	0.53460	0.00000	mCG124907.1	null	null
374491	2.67550	0.61830	0.00000	mCG1036507.2	null	null
902338	2.66330	0.51890	0.00000	mCG129972.1	LOC638099|LOC629893	null
400498	2.62390	0.75150	0.00000	mCG141610.1	null	null
892044	2.60960	0.42700	0.00000	mCG1025954.1	null	null
902277	2.60290	0.68610	0.00000	mCG113668	LOC633719	null
856087	2.58540	0.67150	0.00000	mCG1036442.2	null	null
566733	2.57470	0.93750	0.00000	mCG1026777	LOC639369	null
478281	2.56490	0.98540	0.00010	null	LOC243431	null
657549	2.55990	0.50810	0.00000	mCG1036441.2	LOC620357|LOC637227	null
896839	2.54630	0.47530	0.00000	mCG118864.1	LOC634081|LOC634275	null
497944	2.52610	1.03850	0.00000	null	LOC238418	null
395082	2.49490	0.24860	0.00000	mCG13668.2	LOC238447|LOC544903|LOC544907|Igh-VJ558	immunoglobulin heavy chain (J558 family)
411078	2.49050	0.82620	0.00000	mCG1036502.2|mCG132001.2	LOC434025|LOC243433|LOC628159|LOC635885	null
903525	2.42830	0.89490	0.00000	mCG1036509.2	LOC628516|LOC636646	null
892300	2.42540	1.12620	0.00000	mCG1032189.1	LOC629883	null
650375	2.40130	0.90540	0.00000	mCG142578	LOC213570|LOC636126	null
379964	2.39580	0.52100	0.00000	mCG1025534.1	LOC631129	null
658871	2.37950	0.82770	0.00000	mCG1036438.2|mCG141622.1	Gm1530|Gm1420	gene model 1530, (NCBI)|gene model 1420, (NCBI)
788195	2.35930	0.54530	0.00000	mCG141636.1	LOC545848	null
904211	2.35030	1.20470	0.00000	mCG142561	LOC629906|LOC629908|LOC634791	null
904213	2.34960	0.92070	0.00000	mCG118867	LOC634123	null
316223	2.32080	0.25740	0.00000	mCG1036431.2	ci12	null
658873	2.32030	0.95390	0.00000	mCG126268.2|mCG1036508.2|mCG141637.1|mCG10	Gm1419|Igk-C|Gm459|LOC546244|LOC619635	gene model 1419, (NCBI)|immunoglobulin kappa chain, constant region|gene model 459, (NCBI)
380936	2.28220	0.78480	0.00000	mCG1036436.1	Gm1418	gene model 1418, (NCBI)
823261	2.28190	0.88690	0.00000	mCG126265.2|mCG141634.1	Gm1419|Gm1524|LOC546244	gene model 1419, (NCBI)|gene model 1524, (NCBI)
900977	2.26030	0.91220	0.00000	mCG141630.1	LOC434586	null
894197	2.24640	0.93230	0.00000	mCG116912.1	null	null
557237	2.21780	0.33990	0.00000	mCG146830	LOC634450|LOC625794|LOC624610	null
904212	2.19090	0.91540	0.00000	mCG118865.1	LOC629906|LOC634065|LOC245266	null
372826	2.08430	0.75830	0.00000	mCG141629.1	null	null
910033	1.85080	0.21260	0.00000	mCG131871.2	Igk-V8|LOC545854|Igk-C|LOC243469|Igk-V21|Igk-V28	immunoglobulin kappa chain variable 8 (V8)|immunoglobulin kappa chain, constant region|immunoglobulin kappa chain variable 21 (V21)|immunoglobulin kappa chain variable 2
388152	1.84420	0.96390	0.00000	null	LOC628571	null
305669	1.83780	0.57610	0.00000	mCG142176.1	Igk-V8|LOC637207	immunoglobulin kappa chain variable 8 (V8)

456277	1.78790	0.37940	0.00000	mCG8631.2	Fcgr3a	Fc fragment of IgG, low affinity IIIa, receptor
409578	1.76210	0.53860	0.00000	mCG142168.1|mCG142181.1	LOC620191|LOC637126	null
815506	1.75880	0.68830	0.00020	mCG142169.1	LOC637295|LOC620451	null
895899	1.75600	0.75600	0.00050	mCG127250	LOC638631	null
902343	1.60140	0.51470	0.00000	mCG116911.1	LOC619833|LOC635935|Igh-VJ558	immunoglobulin heavy chain (J558 family)
897263	1.48890	0.24630	0.00000	mCG142552	null	null
910246	1.44630	0.59210	0.00000	mCG142161.1|mCG1036517.2	Igk-V21	immunoglobulin kappa chain variable 21 (V21)
912624	1.37490	0.62600	0.00000	mCG141777	LOC433053|LOC623736|Igl-V1	immunoglobulin lambda chain, variable 1
909884	1.36870	0.41510	0.00000	mCG141776|mCG141779	Igl-V1|2010309G21Rik	immunoglobulin lambda chain, variable 1|RIKEN cDNA 2010309G21 gene
649745	0.92060	0.38690	0.00100	mCG8629.2	Fcgr3	Fc receptor, IgG, low affinity III
774411	0.86170	0.23540	0.00000	mCG144561	Alox5ap	arachidonate 5-lipoxygenase activating protein
465504	0.81420	0.31440	0.00020	mCG8632.2	Fcer1g	Fc receptor, IgE, high affinity I, gamma polypeptide
323019	0.65580	0.08400	0.00000	mCG132542.2	Clec2h	C-type lectin domain family 2, member h

Cytokine and chemokine mediated signaling pathway

PROBE	LOG_Q[090514_J-K_ind.ma2]	VAR[090514_J-K_ind.ma2]	P[090514_J-K_ind.ma2]	Celera Gene ID	Gene_Symbol	Gene_Name
575878	4.39140	0.41670	0.00000	mCG12528.2	Cxcl9	chemokine (C-X-C motif) ligand 9
930152	3.26280	0.49470	0.00000	mCG12531.2	Cxcl10	chemokine (C-X-C motif) ligand 10
624929	2.74780	0.48220	0.00000	mCG2202.1	Cxcr3	chemokine (C-X-C motif) receptor 3
801088	2.46720	0.63340	0.00000	mCG15845.2	Cxcr6	chemokine (C-X-C motif) receptor 6
516139	2.40280	0.34790	0.00000	mCG11684.1	Ccl5	chemokine (C-C motif) ligand 5
907844	1.54020	0.56760	0.00000	mCG8184.2	Ccl2	chemokine (C-C motif) ligand 2
928327	1.26980	0.21370	0.00000	mCG11623.1	Ccl6	chemokine (C-C motif) ligand 6
753313	1.10330	0.55180	0.00000	mCG123600|mCG114938	Ccl8	chemokine (C-C motif) ligand 8
665530	0.55120	0.08910	0.00060	mCG20697.2	Csf2ra	colony stimulating factor 2 receptor, alpha, low-affinity (granulocyte-macrophage)

Cytokine/chemokine mediated immunity

PROBE	LOG_Q[090514_J-K_ind.ma2]	VAR[090514_J-K_ind.ma2]	P[090514_J-K_ind.ma2]	Celera Gene ID	Gene_Symbol	Gene_Name
575878	4.39140	0.41670	0.00000	mCG12528.2	Cxcl9	chemokine (C-X-C motif) ligand 9
930152	3.26280	0.49470	0.00000	mCG12531.2	Cxcl10	chemokine (C-X-C motif) ligand 10
624929	2.74780	0.48220	0.00000	mCG2202.1	Cxcr3	chemokine (C-X-C motif) receptor 3
801088	2.46720	0.63340	0.00000	mCG15845.2	Cxcr6	chemokine (C-X-C motif) receptor 6
516139	2.40280	0.34790	0.00000	mCG11684.1	Ccl5	chemokine (C-C motif) ligand 5
907844	1.54020	0.56760	0.00000	mCG8184.2	Ccl2	chemokine (C-C motif) ligand 2
928327	1.26980	0.21370	0.00000	mCG11623.1	Ccl6	chemokine (C-C motif) ligand 6
753313	1.10330	0.55180	0.00000	mCG123600|mCG114938	Ccl8	chemokine (C-C motif) ligand 8

Immunity and defense

PROBE	LOG_Q[090514_J-K_ind.ma2]	VAR[090514_J-K_ind.ma2]	P[090514_J-K_ind.ma2]	Celera Gene ID	Gene_Symbol	Gene_Name
429239	5.93180	0.93180	0.00000	mCG56372.2	LOC432699	null
550292	5.23590	1.24620	0.00000	mCG4516.2	Gzma	granzyme A
575878	4.39140	0.41670	0.00000	mCG12528.2	Cxcl9	chemokine (C-X-C motif) ligand 9
339582	4.26380	0.89670	0.00000	mCG140410	Igh-1a|LOC544908	immunoglobulin heavy chain 1a (serum IgG2a)
590082	4.13340	0.85190	0.00000	mCG141789.1	Igk-V32	immunoglobulin kappa chain variable 32 (V32)
808065	3.99290	1.80190	0.00000	mCG130744.2|mCG1036427.2	LOC635601|bd2|LOC628027	null
736226	3.97930	1.02240	0.00000	mCG1027522.1|mCG1029835.1	LOC636462	null
401576	3.90870	1.76620	0.00000	mCG132002.2	LOC243439	null
891891	3.85240	1.34100	0.00000	mCG113660.1	LOC633515	null
538665	3.83340	0.58660	0.00000	null	LOC434031|Igk-V38	immunoglobulin kappa chain variable 38(V38)
491036	3.77740	0.84550	0.00000	mCG116913.1	LOC636067	null
699266	3.77400	0.76790	0.00000	mCG1025451.1	null	null
742698	3.74840	0.88410	0.00000	mCG130744.2|mCG1036427.2|mCG1036498.2	LOC635664|LOC635601|bd2|LOC628056|LOC628027	null
493584	3.71590	0.98470	0.00000	mCG142153.1	LOC620105|LOC637047	null
411187	3.70140	0.56530	0.00000	null	LOC620017	null
348117	3.67230	0.45770	0.00000	mCG1036432.2	null	null
857940	3.65340	1.28650	0.00000	mCG1037662.1	LOC630251	null
715331	3.64830	0.51560	0.00000	mCG141621.1	LOC434037	null
901486	3.64130	1.45140	0.00000	mCG68149.2	LOC630331|LOC633472	null
596021	3.63320	1.11870	0.00000	mCG146921.1|mCG141794.1|mCG141802.1	IgM	null
574582	3.58730	1.10810	0.00000	mCG146921.1|mCG141794.1	IgM	null
515370	3.58010	1.03810	0.00000	mCG142162.1	LOC626145|LOC545854	null
444829	3.55200	1.23660	0.00000	mCG141790.1	Gm1067	gene model 1067, (NCBI)
489583	3.43750	0.72290	0.00000	mCG114300.1	null	null
479671	3.42090	0.74830	0.00000	mCG126269.2|mCG1036505.2	gn33|Igk-V34|Igk-V33	immunoglobulin kappa chain variable 34 (V34)|immunoglobulin kappa chain variable 33 (V33)
896758	3.41680	0.08000	0.00000	mCG1037964.1	LOC620216|LOC632220	null
583890	3.36590	0.47530	0.00000	mCG117763.1	LOC634652|LOC382695|LOC436124	null
919480	3.30590	0.97960	0.00000	mCG141784.1	LOC434025	null
675426	3.26900	0.99890	0.00000	mCG128543.1	Igh-1a|LOC382692|LOC628614	immunoglobulin heavy chain 1a (serum IgG2a)
930152	3.26280	0.49470	0.00000	mCG12531.2	Cxcl10	chemokine (C-X-C motif) ligand 10
823783	3.23050	0.56160	0.00000	mCG1036439.2	LOC384413|Igk-V28	immunoglobulin kappa chain variable 28 (V28)
899105	3.22980	0.41100	0.00000	mCG142070	LOC637785	null
739642	3.18500	0.52560	0.00000	mCG141793.1	LOC434026	null
898268	3.16270	0.52810	0.00000	mCG127559.1|mCG114359	LOC640614|LOC632997|LOC636453	null
317586	3.15470	0.98640	0.00010	mCG1025125.1	LOC631518	null
576556	3.14730	1.11630	0.00000	mCG140401	LOC634430	null
902557	3.11220	0.71020	0.00000	mCG127559.1	null	null
843602	3.08470	0.78140	0.00000	mCG118954	LOC638634	null
897561	3.08440	0.54880	0.00000	null	LOC434609	null
483305	3.08070	0.65440	0.00000	null	Gm1419	gene model 1419, (NCBI)
901720	3.07910	0.51480	0.00000	mCG1026782.1	LOC382695|LOC629938|LOC638485	null
523804	3.01550	0.22180	0.00000	mCG1037963.1	LOC632180|LOC432993|LOC620169	null
875823	2.97880	0.64400	0.00000	mCG129378	LOC544903	null
410888	2.95830	0.97940	0.00000	mCG1025140.1	LOC633734	null
672387	2.94750	1.17050	0.00000	mCG114360|mCG1025864.1	null	null
401803	2.93560	0.50790	0.00000	mCG118868.1	LOC634298	null
615665	2.92850	0.88010	0.00000	mCG1050179	D6Mit97|Igk-V28	DNA segment, Chr 6, Massachusetts Institute of Technology 97|immunoglobulin kappa chain variable 28 (V28)
579252	2.92670	0.63600	0.00000	mCG127631.1	Igh-4	immunoglobulin heavy chain 4 (serum IgG1)
832392	2.90250	0.86180	0.00000	null	LOC628127	null
894906	2.89830	0.71310	0.00000	mCG118865.1|mCG114299.1	LOC629884|LOC637000|Igh|Igh-VJ558	immunoglobulin heavy chain complex|immunoglobulin heavy chain (J558 family)
884118	2.80080	1.03780	0.00040	mCG130827	Gzmb	granzyme B

<table>
<tr><th></th><th></th><th></th><th></th><th></th><th></th><th></th></tr>
<tr><td>899101</td><td>2.79970</td><td>1.08000</td><td>0.00000</td><td>mCG129262.1</td><td>Igh-VJ558</td><td>immunoglobulin heavy chain (J558 family)</td></tr>
<tr><td>893766</td><td>2.79370</td><td>0.47810</td><td>0.00000</td><td>mCG116911.1|mCG142745|mCG142069</td><td>LOC619833|LOC382696|LOC637398|LOC637794|VH18</td><td>immunoglobulin heavy chain (J558 family)</td></tr>
<tr><td>894845</td><td>2.79170</td><td>0.66090</td><td>0.00000</td><td>mCG1036509.2|mCG141633.1|mCG141637.1</td><td>LOC628516|LOC636646|LOC636598|LOC628498</td><td>null</td></tr>
<tr><td>621943</td><td>2.75070</td><td>0.43730</td><td>0.00000</td><td>mCG127630.1</td><td>LOC633062</td><td>null</td></tr>
<tr><td>624929</td><td>2.74780</td><td>0.48220</td><td>0.00000</td><td>mCG2202.1</td><td>Cxcr3</td><td>chemokine (C-X-C motif) receptor 3</td></tr>
<tr><td>797351</td><td>2.73710</td><td>0.26570</td><td>0.00000</td><td>mCG118871.1|mCG114299.1|mCG140407</td><td>Igh-1a|LOC634338|LOC629884|LOC629871|LOC63422</td><td>immunoglobulin heavy chain 1a (serum IgG2a)|immunoglobulin heavy chain complex|immunoglobulin heavy chain (J558 family)</td></tr>
<tr><td>721976</td><td>2.72350</td><td>0.78620</td><td>0.00000</td><td>mCG141796.1</td><td>LOC545847</td><td>null</td></tr>
<tr><td>892758</td><td>2.68780</td><td>0.73590</td><td>0.00000</td><td>mCG118870.1</td><td>LOC634541</td><td>null</td></tr>
<tr><td>685068</td><td>2.68190</td><td>0.96900</td><td>0.00000</td><td>mCG131876.2</td><td>null</td><td>null</td></tr>
<tr><td>902558</td><td>2.67750</td><td>0.53460</td><td>0.00000</td><td>mCG124907.1</td><td>null</td><td>null</td></tr>
<tr><td>374491</td><td>2.67550</td><td>0.61830</td><td>0.00000</td><td>mCG1036507.2</td><td>null</td><td>null</td></tr>
<tr><td>902338</td><td>2.66330</td><td>0.51890</td><td>0.00000</td><td>mCG129972.1</td><td>LOC638099|LOC629893</td><td>null</td></tr>
<tr><td>712519</td><td>2.65430</td><td>0.25200</td><td>0.00000</td><td>null</td><td>H2-Q8</td><td>histocompatibility 2, Q region locus 8</td></tr>
<tr><td>400498</td><td>2.62390</td><td>0.75150</td><td>0.00000</td><td>mCG141610.1</td><td>null</td><td>null</td></tr>
<tr><td>586296</td><td>2.61800</td><td>0.56980</td><td>0.00010</td><td>mCG146987</td><td>Gbp1</td><td>guanylate nucleotide binding protein 1</td></tr>
<tr><td>892044</td><td>2.60960</td><td>0.42700</td><td>0.00000</td><td>mCG1025954.1</td><td>null</td><td>null</td></tr>
<tr><td>902277</td><td>2.60290</td><td>0.68610</td><td>0.00000</td><td>mCG113668</td><td>LOC633719</td><td>null</td></tr>
<tr><td>320903</td><td>2.58600</td><td>0.32230</td><td>0.00000</td><td>null</td><td>H2-Q1</td><td>histocompatibility 2, Q region locus 1</td></tr>
<tr><td>856087</td><td>2.58540</td><td>0.67150</td><td>0.00000</td><td>mCG1036442.2</td><td>null</td><td>null</td></tr>
<tr><td>566733</td><td>2.57470</td><td>0.93750</td><td>0.00000</td><td>mCG1026777</td><td>LOC639369</td><td>null</td></tr>
<tr><td>478281</td><td>2.56490</td><td>0.98540</td><td>0.00010</td><td>null</td><td>LOC243431</td><td>null</td></tr>
<tr><td>431279</td><td>2.56150</td><td>0.38030</td><td>0.00000</td><td>mCG113208.1</td><td>H2-Ea|H2-Aa</td><td>histocompatibility 2, class II antigen E alpha|histocompatibility 2, class II antigen A, alpha</td></tr>
<tr><td>657549</td><td>2.55990</td><td>0.50810</td><td>0.00000</td><td>mCG1036441.2</td><td>LOC620357|LOC637227</td><td>null</td></tr>
<tr><td>896839</td><td>2.54630</td><td>0.47530</td><td>0.00000</td><td>mCG118864.1</td><td>LOC634081|LOC634275</td><td>null</td></tr>
<tr><td>745275</td><td>2.54470</td><td>0.18670</td><td>0.00000</td><td>mCG23016.2</td><td>H2-DMa</td><td>histocompatibility 2, class II, locus DMa</td></tr>
<tr><td>497944</td><td>2.52610</td><td>1.03850</td><td>0.00000</td><td>null</td><td>LOC238418</td><td>null</td></tr>
<tr><td>395082</td><td>2.49490</td><td>0.24860</td><td>0.00000</td><td>mCG13668.2</td><td>LOC238447|LOC544903|LOC544907|Igh-VJ558</td><td>immunoglobulin heavy chain (J558 family)</td></tr>
<tr><td>411078</td><td>2.49050</td><td>0.82620</td><td>0.00000</td><td>mCG1036502.2|mCG132001.2</td><td>LOC434025|LOC243433|LOC628159|LOC635885</td><td>null</td></tr>
<tr><td>801088</td><td>2.46720</td><td>0.63340</td><td>0.00000</td><td>mCG15845.2</td><td>Cxcr6</td><td>chemokine (C-X-C motif) receptor 6</td></tr>
<tr><td>903525</td><td>2.42830</td><td>0.89490</td><td>0.00000</td><td>mCG1036509.2</td><td>LOC628516|LOC636646</td><td>null</td></tr>
<tr><td>892300</td><td>2.42540</td><td>1.12620</td><td>0.00000</td><td>mCG1032189.1</td><td>LOC629883</td><td>null</td></tr>
<tr><td>516139</td><td>2.40280</td><td>0.34790</td><td>0.00000</td><td>mCG11684.1</td><td>Ccl5</td><td>chemokine (C-C motif) ligand 5</td></tr>
<tr><td>650375</td><td>2.40130</td><td>0.90540</td><td>0.00000</td><td>mCG142578</td><td>LOC213570|LOC636126</td><td>null</td></tr>
<tr><td>379964</td><td>2.39580</td><td>0.52100</td><td>0.00000</td><td>mCG1025534.1</td><td>LOC631129</td><td>null</td></tr>
<tr><td>658871</td><td>2.37950</td><td>0.82770</td><td>0.00000</td><td>mCG1036438.2|mCG141622.1</td><td>Gm1530|Gm1420</td><td>gene model 1530, (NCBI)|gene model 1420, (NCBI)</td></tr>
<tr><td>847876</td><td>2.37220</td><td>0.26840</td><td>0.00000</td><td>mCG122229</td><td>Gbp2</td><td>guanylate nucleotide binding protein 2</td></tr>
<tr><td>788195</td><td>2.35930</td><td>0.54530</td><td>0.00000</td><td>mCG141636.1</td><td>LOC545848</td><td>null</td></tr>
<tr><td>904211</td><td>2.35030</td><td>1.20470</td><td>0.00000</td><td>mCG142561</td><td>LOC629906|LOC629908|LOC634791</td><td>null</td></tr>
<tr><td>904213</td><td>2.34960</td><td>0.92070</td><td>0.00000</td><td>mCG118867</td><td>LOC634123</td><td>null</td></tr>
<tr><td>316223</td><td>2.32080</td><td>0.25740</td><td>0.00000</td><td>mCG1036431.2</td><td>ci12</td><td>null</td></tr>
<tr><td>658873</td><td>2.32030</td><td>0.95390</td><td>0.00000</td><td>mCG126268.2|mCG1036508.2|mCG141637.1|mCG10</td><td>Gm1419|Igk-C|Gm459|LOC546244|LOC619635</td><td>gene model 1419, (NCBI)|immunoglobulin kappa chain, constant region|gene model 459, (NCBI)</td></tr>
<tr><td>380936</td><td>2.28220</td><td>0.78480</td><td>0.00000</td><td>mCG1036436.1</td><td>Gm1418</td><td>gene model 1418, (NCBI)</td></tr>
<tr><td>823261</td><td>2.28190</td><td>0.88690</td><td>0.00000</td><td>mCG126265.2|mCG141634.1</td><td>Gm1419|Gm1524|LOC546244</td><td>gene model 1419, (NCBI)|gene model 1524, (NCBI)</td></tr>
<tr><td>637912</td><td>2.27350</td><td>0.18870</td><td>0.00000</td><td>mCG132704.1</td><td>H2-Ab1|LOC641240</td><td>histocompatibility 2, class II antigen A, beta 1</td></tr>
<tr><td>900977</td><td>2.26030</td><td>0.91220</td><td>0.00000</td><td>mCG141630.1</td><td>LOC434586</td><td>null</td></tr>
<tr><td>904963</td><td>2.25850</td><td>0.40890</td><td>0.00000</td><td>mCG132703.2|mCG132689.1</td><td>H2-DMb2|H2-DMb1</td><td>histocompatibility 2, class II, locus Mb2|histocompatibility 2, class II, locus Mb1</td></tr>
<tr><td>775978</td><td>2.24760</td><td>0.20400</td><td>0.00000</td><td>mCG6027.2</td><td>Cd74</td><td>CD74 antigen (invariant polypeptide of major histocompatibility complex, class II antigen-associated)</td></tr>
<tr><td>894197</td><td>2.24640</td><td>0.93230</td><td>0.00000</td><td>mCG116912.1</td><td>null</td><td>null</td></tr>
<tr><td>920968</td><td>2.22910</td><td>0.24630</td><td>0.00000</td><td>mCG9940.2</td><td>H2-Eb1</td><td>histocompatibility 2, class II antigen E beta</td></tr>
<tr><td>557237</td><td>2.21780</td><td>0.33990</td><td>0.00000</td><td>mCG146830</td><td>LOC634450|LOC625794|LOC624610</td><td>null</td></tr>
<tr><td>904212</td><td>2.19090</td><td>0.91540</td><td>0.00000</td><td>mCG118865.1</td><td>LOC629906|LOC634065|LOC245266</td><td>null</td></tr>
<tr><td>643417</td><td>2.17360</td><td>0.64740</td><td>0.00000</td><td>mCG124141</td><td>null</td><td>null</td></tr>
<tr><td>372826</td><td>2.08430</td><td>0.75830</td><td>0.00000</td><td>mCG141629.1</td><td>null</td><td>null</td></tr>
<tr><td>522474</td><td>2.05370</td><td>0.62000</td><td>0.00000</td><td>mCG126563.1</td><td>C2ta</td><td>class II transactivator</td></tr>
<tr><td>873670</td><td>2.02300</td><td>0.22280</td><td>0.00000</td><td>mCG23012.2</td><td>Tap1</td><td>transporter 1, ATP-binding cassette, sub-family B (MDR/TAP)</td></tr>
<tr><td>780747</td><td>1.99790</td><td>0.49670</td><td>0.00010</td><td>mCG127285.2</td><td>LOC636147|Cd8a</td><td>CD8 antigen, alpha chain</td></tr>
<tr><td>742215</td><td>1.96190</td><td>0.23130</td><td>0.00000</td><td>mCG8696.2</td><td>Tcra|LOC545051|A430107P09Rik</td><td>T-cell receptor alpha chain|RIKEN cDNA A430107P09 gene</td></tr>
<tr><td>447264</td><td>1.95530</td><td>0.48700</td><td>0.00030</td><td>mCG11758.2</td><td>Ctsw</td><td>cathepsin W</td></tr>
<tr><td>910033</td><td>1.85080</td><td>0.21260</td><td>0.00000</td><td>mCG131871.2</td><td>Igk-V8|LOC545854|Igk-C|LOC243469|Igk-V21|Igk-V28</td><td>immunoglobulin kappa chain variable 8 (V8)|immunoglobulin kappa chain, constant region|immunoglobulin kappa chain variable 21 (V21)|immunoglobulin kappa chain variable 2</td></tr>
<tr><td>388152</td><td>1.84420</td><td>0.96390</td><td>0.00000</td><td>null</td><td>LOC628571</td><td>null</td></tr>
<tr><td>305669</td><td>1.83780</td><td>0.57610</td><td>0.00000</td><td>mCG142176.1</td><td>Igk-V8|LOC637207</td><td>immunoglobulin kappa chain variable 8 (V8)</td></tr>
<tr><td>616423</td><td>1.80620</td><td>0.42350</td><td>0.00000</td><td>mCG20328.2</td><td>5830443L24Rik</td><td>RIKEN cDNA 5830443L24 gene</td></tr>
<tr><td>456277</td><td>1.78790</td><td>0.37940</td><td>0.00000</td><td>mCG8631.2</td><td>Fcgr3a</td><td>Fc fragment of IgG, low affinity IIIa, receptor</td></tr>
<tr><td>840423</td><td>1.78240</td><td>0.29170</td><td>0.00000</td><td>mCG1551.2</td><td>Cd3g</td><td>CD3 antigen, gamma polypeptide</td></tr>
<tr><td>405120</td><td>1.77250</td><td>0.22730</td><td>0.00000</td><td>mCG21119.2</td><td>Gbp4</td><td>guanylate nucleotide binding protein 4</td></tr>
<tr><td>409578</td><td>1.76210</td><td>0.53860</td><td>0.00000</td><td>mCG142168.1|mCG142181.1</td><td>LOC620191|LOC637126</td><td>null</td></tr>
<tr><td>815506</td><td>1.75880</td><td>0.68830</td><td>0.00020</td><td>mCG142169.1</td><td>LOC637295|LOC620451</td><td>null</td></tr>
<tr><td>895899</td><td>1.75600</td><td>0.75600</td><td>0.00050</td><td>mCG127250</td><td>LOC638631</td><td>null</td></tr>
<tr><td>453230</td><td>1.74770</td><td>0.32150</td><td>0.00000</td><td>mCG1536.1</td><td>Cd3d</td><td>CD3 antigen, delta polypeptide</td></tr>
<tr><td>913926</td><td>1.72950</td><td>0.51800</td><td>0.00000</td><td>mCG141961</td><td>9830147J24Rik</td><td>RIKEN cDNA 9830147J24 gene</td></tr>
<tr><td>613784</td><td>1.67590</td><td>0.37020</td><td>0.00000</td><td>mCG17747.2</td><td>Zap70</td><td>zeta-chain (TCR) associated protein kinase</td></tr>
<tr><td>902343</td><td>1.60140</td><td>0.51470</td><td>0.00000</td><td>mCG116911.1</td><td>LOC619833|LOC635935|Igh-VJ558</td><td>immunoglobulin heavy chain (J558 family)</td></tr>
<tr><td>501966</td><td>1.57600</td><td>0.44910</td><td>0.00000</td><td>mCG147911</td><td>B2m</td><td>beta-2 microglobulin</td></tr>
<tr><td>907844</td><td>1.54020</td><td>0.56760</td><td>0.00000</td><td>mCG8184.2</td><td>Ccl2</td><td>chemokine (C-C motif) ligand 2</td></tr>
<tr><td>897263</td><td>1.48890</td><td>0.24630</td><td>0.00000</td><td>mCG142552</td><td>null</td><td>null</td></tr>
<tr><td>910246</td><td>1.44630</td><td>0.59210</td><td>0.00000</td><td>mCG142161.1|mCG1036517.2</td><td>Igk-V21</td><td>immunoglobulin kappa chain variable 21 (V21)</td></tr>
<tr><td>917567</td><td>1.43150</td><td>0.27250</td><td>0.00000</td><td>mCG15923.1</td><td>Aif1</td><td>allograft inflammatory factor 1</td></tr>
<tr><td>912624</td><td>1.37490</td><td>0.62600</td><td>0.00000</td><td>mCG141777</td><td>LOC433053|LOC623736|Igl-V1</td><td>immunoglobulin lambda chain, variable 1</td></tr>
<tr><td>909884</td><td>1.36870</td><td>0.41510</td><td>0.00000</td><td>mCG141776|mCG141779</td><td>Igl-V1|2010309G21Rik</td><td>immunoglobulin lambda chain, variable 1|RIKEN cDNA 2010309G21 gene</td></tr>
<tr><td>909409</td><td>1.35240</td><td>0.42110</td><td>0.00000</td><td>mCG9333.2</td><td>C1qb</td><td>complement component 1, q subcomponent, beta polypeptide</td></tr>
<tr><td>607896</td><td>1.33990</td><td>0.28910</td><td>0.00000</td><td>mCG21470.1</td><td>Ifit1</td><td>interferon-induced protein with tetratricopeptide repeats 1</td></tr>
<tr><td>314727</td><td>1.32380</td><td>0.32000</td><td>0.00000</td><td>mCG10007.2</td><td>Cd48</td><td>CD48 antigen</td></tr>
<tr><td>351046</td><td>1.32030</td><td>0.18470</td><td>0.00000</td><td>mCG132214.1</td><td>Clec4a1</td><td>C-type lectin domain family 4, member a1</td></tr>
<tr><td>315737</td><td>1.30020</td><td>0.48310</td><td>0.00000</td><td>mCG3553.3</td><td>Selpl</td><td>selectin, platelet (p-selectin) ligand</td></tr>
<tr><td>928327</td><td>1.26980</td><td>0.21370</td><td>0.00000</td><td>mCG11623.1</td><td>Ccl6</td><td>chemokine (C-C motif) ligand 6</td></tr>
<tr><td>454874</td><td>1.23930</td><td>0.16550</td><td>0.00000</td><td>mCG6391.1</td><td>Ptpn22</td><td>protein tyrosine phosphatase, non-receptor type 22 (lymphoid)</td></tr>
<tr><td>736887</td><td>1.21900</td><td>0.11950</td><td>0.00000</td><td>mCG22139.1</td><td>Nkg7</td><td>natural killer cell group 7 sequence</td></tr>
<tr><td>737611</td><td>1.16720</td><td>0.29130</td><td>0.00000</td><td>null</td><td>Lck</td><td>lymphocyte protein tyrosine kinase</td></tr>
<tr><td>355529</td><td>1.15290</td><td>0.20110</td><td>0.00000</td><td>mCG132215.1</td><td>Clec4a3</td><td>C-type lectin domain family 4, member a3</td></tr>
<tr><td>753313</td><td>1.10330</td><td>0.55180</td><td>0.00000</td><td>mCG123600|mCG114938</td><td>Ccl8</td><td>chemokine (C-C motif) ligand 8</td></tr>
<tr><td>812906</td><td>1.08450</td><td>0.38480</td><td>0.00000</td><td>mCG144019</td><td>Saa2</td><td>serum amyloid A 2</td></tr>
</table>

440084	1.07700	0.18760	0.00000	mCG9108.1	Cd274	CD274 antigen
812669	1.06680	0.25910	0.00010	mCG3074.1	Cd1d1	CD1d1 antigen
604623	1.06550	0.09790	0.00000	null	Parp3	poly (ADP-ribose) polymerase family, member 3
524163	1.04600	0.18300	0.00000	mCG9327.1	C1qa	complement component 1, q subcomponent, alpha polypeptide
732899	1.04450	0.23180	0.00000	mCG141548	Oasl2	2'-5' oligoadenylate synthetase-like 2
374617	1.04100	0.22850	0.00000	mCG3049.3	Lyzs	lysozyme
897539	1.03280	0.07740	0.00000	mCG142248.1	LOC633551|C4|Slp	complement component 4 (within H-2S)|sex-limited protein
675339	1.02480	0.35290	0.00000	mCG9326.1	C1qg	complement component 1, q subcomponent, gamma polypeptide
925708	0.98980	0.20940	0.00000	mCG22590.1	Ifitm3	interferon induced transmembrane protein 3
922460	0.97840	0.09980	0.00000	mCG13768.1	Irf1	interferon regulatory factor 1
612867	0.97300	0.41510	0.00060	mCG128047.2	Gimap4	GTPase, IMAP family member 4
800022	0.96700	0.18650	0.00000	mCG22805.2	Tyrobp	TYRO protein tyrosine kinase binding protein
908953	0.94240	0.09150	0.00000	mCG11606.2	B2m	beta-2 microglobulin
649745	0.92060	0.38690	0.00100	mCG8629.2	Fcgr3	Fc receptor, IgG, low affinity III
923823	0.91860	0.24270	0.00000	mCG1253.1	Pglyrp1	peptidoglycan recognition protein 1
692814	0.90880	0.13390	0.00000	mCG21986.3	Samhd1	SAM domain and HD domain, 1
638437	0.88810	0.06670	0.00000	mCG132391.2	H2-T9|H2-T17|H2-T22|H2-T10	histocompatibility 2, T region locus 9|histocompatibility 2, T region locus 17|histocompatibility 2, T region locus 22|histocompatibility 2, T region locus 10
847591	0.87240	0.18830	0.00000	mCG134313.1	Tapbpl	TAP binding protein-like
774411	0.86170	0.23540	0.00000	mCG144561	Alox5ap	arachidonate 5-lipoxygenase activating protein
454213	0.85610	0.31890	0.00000	mCG3047.2	Lzp-s	P lysozyme structural
930739	0.84530	0.22190	0.00000	mCG141466	C1r	complement component 1, r subcomponent
465504	0.81420	0.31440	0.00020	mCG8632.2	Fcer1g	Fc receptor, IgE, high affinity I, gamma polypeptide
558496	0.79940	0.13770	0.00000	mCG12223.1	H2-T23	histocompatibility 2, T region locus 23
414909	0.78410	0.27060	0.00020	mCG6318.1	Ifi203	interferon activated gene 203
928306	0.77450	0.10840	0.00000	mCG23013.2	Tap2	transporter 2, ATP-binding cassette, sub-family B (MDR/TAP)
766362	0.74930	0.14560	0.00000	mCG125800.1	Fyn	Fyn proto-oncogene
334297	0.71020	0.20460	0.00010	null	Cd99	CD99 antigen
912733	0.67340	0.08560	0.00000	mCG129013.1	Cd47	CD47 antigen (Rh-related antigen, integrin-associated signal transducer)
323019	0.65580	0.08400	0.00000	mCG132542.2	Clec2h	C-type lectin domain family 2, member h
916013	0.64810	0.18630	0.00040	mCG11072.3	Lsp1	lymphocyte specific 1
675921	0.59630	0.06190	0.00000	mCG120898.1	LOC633897	null
351041	0.55290	0.08600	0.00040	mCG129426.1	Cd9	CD9 antigen
665530	0.55120	0.08910	0.00060	mCG20697.2	Csf2ra	colony stimulating factor 2 receptor, alpha, low-affinity (granulocyte-macrophage)

Interferon-mediated immunity

PROBE	LOG_Q[090514_J-K_ind.ma2]	VAR[090514_J-K_ind.ma2]	P[090514_J-K_ind.ma2]	Celera Gene ID	Gene_Symbol	Gene_Name
575878	4.39140	0.41670	0.00000	mCG12528.2	Cxcl9	chemokine (C-X-C motif) ligand 9
930152	3.26280	0.49470	0.00000	mCG12531.2	Cxcl10	chemokine (C-X-C motif) ligand 10
586296	2.61800	0.56980	0.00010	mCG146987	Gbp1	guanylate nucleotide binding protein 1
847876	2.37220	0.26840	0.00000	mCG122229	Gbp2	guanylate nucleotide binding protein 2
643417	2.17360	0.64740	0.00000	mCG124141	null	null
616423	1.80620	0.42350	0.00000	mCG20328.2	5830443L24Rik	RIKEN cDNA 5830443L24 gene
405120	1.77250	0.22730	0.00000	mCG21119.2	Gbp4	guanylate nucleotide binding protein 4
913926	1.72950	0.51800	0.00000	mCG141961	9830147J24Rik	RIKEN cDNA 9830147J24 gene
607896	1.33990	0.28910	0.00000	mCG21470.1	Ifit1	interferon-induced protein with tetratricopeptide repeats 1
732899	1.04450	0.23180	0.00000	mCG141548	Oasl2	2'-5' oligoadenylate synthetase-like 2
922460	0.97840	0.09980	0.00000	mCG13768.1	Irf1	interferon regulatory factor 1
414909	0.78410	0.27060	0.00020	mCG6318.1	Ifi203	interferon activated gene 203

MHCI-mediated immunity

PROBE	LOG_Q[090514_J-K_ind.ma2]	VAR[090514_J-K_ind.ma2]	P[090514_J-K_ind.ma2]	Celera Gene ID	Gene_Symbol	Gene_Name
712519	2.65430	0.25200	0.00000	null	H2-Q8	histocompatibility 2, Q region locus 8
320903	2.58600	0.32230	0.00000	null	H2-Q1	histocompatibility 2, Q region locus 1
780747	1.99790	0.49670	0.00010	mCG127285.2	LOC636147|Cd8a	CD8 antigen, alpha chain
447264	1.95530	0.48700	0.00030	mCG11758.2	Ctsw	cathepsin W
501966	1.57600	0.44910	0.00000	mCG147911	B2m	beta-2 microglobulin
908953	0.94240	0.09150	0.00000	mCG11606.2	B2m	beta-2 microglobulin
638437	0.88810	0.06670	0.00000	mCG132391.2	H2-T9|H2-T17|H2-T22|H2-T10	histocompatibility 2, T region locus 9|histocompatibility 2, T region locus 17|histocompatibility 2, T region locus 22|histocompatibility 2, T region locus 10
847591	0.87240	0.18830	0.00000	mCG134313.1	Tapbpl	TAP binding protein-like
558496	0.79940	0.13770	0.00000	mCG12223.1	H2-T23	histocompatibility 2, T region locus 23
675921	0.59630	0.06190	0.00000	mCG120898.1	LOC633897	null

MHCII-mediated immunity

PROBE	LOG_Q[090514_J-K_ind.ma2]	VAR[090514_J-K_ind.ma2]	P[090514_J-K_ind.ma2]	Celera Gene ID	Gene_Symbol	Gene_Name
431279	2.56150	0.38030	0.00000	mCG113208.1	H2-Ea|H2-Aa	histocompatibility 2, class II antigen E alpha|histocompatibility 2, class II antigen A, alpha
745275	2.54470	0.18670	0.00000	mCG23016.2	H2-DMa	histocompatibility 2, class II, locus DMa
637912	2.27350	0.18870	0.00000	mCG132704.1	H2-Ab1|LOC641120	histocompatibility 2, class II antigen A, beta 1
904963	2.25850	0.40890	0.00000	mCG132703.2|mCG132689.1	H2-DMb2|H2-DMb1	histocompatibility 2, class II, locus Mb2|histocompatibility 2, class II, locus Mb1
775978	2.24760	0.20400	0.00000	mCG6027.2	Cd74	CD74 antigen (invariant polypeptide of major histocompatibility complex, class II antigen-associated)
920968	2.22910	0.24630	0.00000	mCG9940.2	H2-Eb1	histocompatibility 2, class II antigen E beta
522474	2.05370	0.62000	0.00000	mCG126563.1	C2ta	class II transactivator

Macrophage-mediated immunity

PROBE	LOG_Q[090514_J-K_ind.ma2]	VAR[090514_J-K_ind.ma2]	P[090514_J-K_ind.ma2]	Celera Gene ID	Gene_Symbol	Gene_Name
575878	4.39140	0.41670	0.00000	mCG12528.2	Cxcl9	chemokine (C-X-C motif) ligand 9
930152	3.26280	0.49470	0.00000	mCG12531.2	Cxcl10	chemokine (C-X-C motif) ligand 10
586296	2.61800	0.56980	0.00010	mCG146987	Gbp1	guanylate nucleotide binding protein 1
847876	2.37220	0.26840	0.00000	mCG122229	Gbp2	guanylate nucleotide binding protein 2
616423	1.80620	0.42350	0.00000	mCG20328.2	5830443L24Rik	RIKEN cDNA 5830443L24 gene
456277	1.78790	0.37940	0.00000	mCG8631.2	Fcgr3a	Fc fragment of IgG, low affinity IIIa, receptor
405120	1.77250	0.22730	0.00000	mCG21119.2	Gbp4	guanylate nucleotide binding protein 4
913926	1.72950	0.51800	0.00000	mCG141961	9830147J24Rik	RIKEN cDNA 9830147J24 gene
351046	1.32030	0.18470	0.00000	mCG132214.1	Clec4a1	C-type lectin domain family 4, member a1
355529	1.15290	0.20110	0.00000	mCG132215.1	Clec4a3	C-type lectin domain family 4, member a3
649745	0.92060	0.38690	0.00100	mCG8629.2	Fcgr3	Fc receptor, IgG, low affinity III
774411	0.86170	0.23540	0.00000	mCG144561	Alox5ap	arachidonate 5-lipoxygenase activating protein

Natural killer cell mediated immunity

PROBE	LOG_Q[090514_J-K_ind.ma2]	VAR[090514_J-K_ind.ma2]	P[090514_J-K_ind.ma2]	Celera Gene ID	Gene_Symbol	Gene_Name
550292	5.23590	1.24620	0.00000	mCG4516.2	Gzma	granzyme A
447264	1.95530	0.48700	0.00030	mCG11758.2	Ctsw	cathepsin W

456277	1.78790	0.37940	0.00000	mCG8631.2	Fcgr3a	Fc fragment of IgG, low affinity IIIa, receptor
736887	1.21900	0.11950	0.00000	mCG22139.1	Nkg7	natural killer cell group 7 sequence
800022	0.96700	0.18650	0.00000	mCG22805.2	Tyrobp	TYRO protein tyrosine kinase binding protein
649745	0.92060	0.38690	0.00100	mCG8629.2	Fcgr3	Fc receptor, IgG, low affinity III
323019	0.65580	0.08400	0.00000	mCG132542.2	Clec2h	C-type lectin domain family 2, member h

T-cell mediated immunity

<table>
<tr><th>PROBE</th><th>LOG_Q[090514_J-K_ind.ma2]</th><th>VAR[090514_J-K_ind.ma2]</th><th>P[090514_J-K_ind.ma2]</th><th>Celera Gene ID</th><th>Gene_Symbol</th><th>Gene_Name</th></tr>
<tr><td>550292</td><td>5.23590</td><td>1.24620</td><td>0.00000</td><td>mCG4516.2</td><td>Gzma</td><td>granzyme A</td></tr>
<tr><td>712519</td><td>2.65430</td><td>0.25200</td><td>0.00000</td><td>null</td><td>H2-Q8</td><td>histocompatibility 2, Q region locus 8</td></tr>
<tr><td>320903</td><td>2.58600</td><td>0.32230</td><td>0.00000</td><td>null</td><td>H2-Q1</td><td>histocompatibility 2, Q region locus 1</td></tr>
<tr><td>431279</td><td>2.56150</td><td>0.38030</td><td>0.00000</td><td>mCG113208.1</td><td>H2-Ea|H2-Aa</td><td>histocompatibility 2, class II antigen E alpha|histocompatibility 2, class II antigen A, alpha</td></tr>
<tr><td>745275</td><td>2.54470</td><td>0.18670</td><td>0.00000</td><td>mCG23016.2</td><td>H2-DMa</td><td>histocompatibility 2, class II, locus DMa</td></tr>
<tr><td>637912</td><td>2.27350</td><td>0.18870</td><td>0.00000</td><td>mCG132704.1</td><td>H2-Ab1|LOC641240</td><td>histocompatibility 2, class II antigen A, beta 1</td></tr>
<tr><td>904963</td><td>2.25850</td><td>0.40890</td><td>0.00000</td><td>mCG132703.2|mCG132689.1</td><td>H2-DMb2|H2-DMb1</td><td>histocompatibility 2, class II, locus Mb2|histocompatibility 2, class II, locus Mb1</td></tr>
<tr><td>775978</td><td>2.24760</td><td>0.20400</td><td>0.00000</td><td>mCG6027.2</td><td>Cd74</td><td>CD74 antigen (invariant polypeptide of major histocompatibility complex, class II antigen-associated)</td></tr>
<tr><td>920968</td><td>2.22910</td><td>0.24630</td><td>0.00000</td><td>mCG9940.2</td><td>H2-Eb1</td><td>histocompatibility 2, class II antigen E beta</td></tr>
<tr><td>522474</td><td>2.05370</td><td>0.62000</td><td>0.00000</td><td>mCG126563.1</td><td>C2ta</td><td>class II transactivator</td></tr>
<tr><td>780747</td><td>1.99790</td><td>0.49670</td><td>0.00010</td><td>mCG127285.2</td><td>LOC636147|Cd8a</td><td>CD8 antigen, alpha chain</td></tr>
<tr><td>742215</td><td>1.96190</td><td>0.23130</td><td>0.00000</td><td>mCG8696.2</td><td>Tcra|LOC545051|A430107P09Rik</td><td>T-cell receptor alpha chain|RIKEN cDNA A430107P09 gene</td></tr>
<tr><td>447264</td><td>1.95530</td><td>0.48700</td><td>0.00030</td><td>mCG11758.2</td><td>Ctsw</td><td>cathepsin W</td></tr>
<tr><td>840423</td><td>1.78240</td><td>0.29170</td><td>0.00000</td><td>mCG1551.2</td><td>Cd3g</td><td>CD3 antigen, gamma polypeptide</td></tr>
<tr><td>453230</td><td>1.74770</td><td>0.32150</td><td>0.00000</td><td>mCG1536.1</td><td>Cd3d</td><td>CD3 antigen, delta polypeptide</td></tr>
<tr><td>501966</td><td>1.57600</td><td>0.44910</td><td>0.00000</td><td>mCG147911</td><td>B2m</td><td>beta-2 microglobulin</td></tr>
<tr><td>440084</td><td>1.07700</td><td>0.18760</td><td>0.00000</td><td>mCG9108.1</td><td>Cd274</td><td>CD274 antigen</td></tr>
<tr><td>812669</td><td>1.06680</td><td>0.25910</td><td>0.00010</td><td>mCG3074.1</td><td>Cd1d1</td><td>CD1d1 antigen</td></tr>
<tr><td>908953</td><td>0.94240</td><td>0.09150</td><td>0.00000</td><td>mCG11606.2</td><td>B2m</td><td>beta-2 microglobulin</td></tr>
<tr><td>638437</td><td>0.88810</td><td>0.06670</td><td>0.00000</td><td>mCG132391.2</td><td>H2-T9|H2-T17|H2-T22|H2-T10</td><td>histocompatibility 2, T region locus 9|histocompatibility 2, T region locus 17|histocompatibility 2, T region locus 22|histocompatibility 2, T region locus 10</td></tr>
<tr><td>847591</td><td>0.87240</td><td>0.18830</td><td>0.00000</td><td>mCG134313.1</td><td>Tapbpl</td><td>TAP binding protein-like</td></tr>
<tr><td>558496</td><td>0.79940</td><td>0.13770</td><td>0.00000</td><td>mCG12223.1</td><td>H2-T23</td><td>histocompatibility 2, T region locus 23</td></tr>
<tr><td>334297</td><td>0.71020</td><td>0.20460</td><td>0.00010</td><td>null</td><td>Cd99</td><td>CD99 antigen</td></tr>
<tr><td>675921</td><td>0.59630</td><td>0.06190</td><td>0.00000</td><td>mCG120898.1</td><td>LOC633897</td><td>null</td></tr>
</table>
