# Supplementary material for: The Microbiota Mediates Pathogen Clearance from the Gut Lumen after Non-Typhoidal Salmonella Diarrhea
Source: PLoS Pathog. 2010 Sep 9;6(9):e1001097. doi: 10.1371/journal.ppat.1001097 (PMC2936549; doi:10.1371/journal.ppat.1001097)
Supplement: Table S4 — Quantitative microbiota analysis by 454 amplicon sequencing. (0.39 MB PDF) [file ppat.1001097.s013.pdf]

Table S4. Quantitative Microbiota analysis by 454 amplicon sequencing

	Chao 1	Shannon (H)	E
S. tm ^{att} L (d.2)	49±15	0.4±0.4	0.1±0.1
S. tm ^{att} L (d.40)	62±14	0.8±0.3	0.2±0.1
S. tm ^{att} L (d.83)	53±31	0.4±0.3	0.1±0
S. tm ^{att} L /C	504±46	4.6±0.1	0.8±0
C	423	4.5	0.8

***** The **Shannon-index** (H') is a measure of species diversity taking into account the number of species and the evenness of the species. i =number of species: p_i= relative abundance of species i; H' is maximal if all species are present at equal abundance (H^{max} = log(1/i); i = number of species).

$$H' = -\sum_i p_i \cdot \ln p_i$$

# Species Evenness: H'/H^{max}; (0-1) An E value of 1 means that the abundance of all species is the same.

‡ The **Chao1 estimato**r estimates total species richness as

$$SChao1 = Sobs + \frac{n1}{2n2}$$

where S_{obs} is the number of observed species, n_1 is the number of singletons (species captured once), and n_2 is the number of doubletons (species captured twice) [15].

Supplemental References

1. Hoiseth SK, Stocker BA (1981) Aromatic-dependent *Salmonella typhimurium* are non-virulent and effective as live vaccines. Nature 291: 238-239.

2. Hapfelmeier S, Ehrbar K, Stecher B, Barthel M, Kremer M, et al. (2004) Role of the Salmonella Pathogenicity Island 1 Effector Proteins SipA, SopB, SopE, and SopE2 in Salmonella enterica Subspecies 1 Serovar Typhimurium Colitis in Streptomycin-Pretreated Mice. Infect Immun 72: 795-809.

3. Ilg K, Endt K, Misselwitz B, Stecher B, Aebi M, et al. (2009) O-antigen-negative Salmonella enterica serovar Typhimurium is attenuated in intestinal colonization but elicits colitis in streptomycin-treated mice. Infect Immun 77: 2568-2575.

4. Stecher B, Barthel M, Schlumberger MC, Haberli L, Rabsch W, et al. (2008) Motility allows S. Typhimurium to benefit from the mucosal defence. Cell Microbiol 10: 1166-1180.

5. Suar M, Jantsch J, Hapfelmeier S, Kremer M, Stallmach T, et al. (2006) Virulence of broad- and narrow-host-range Salmonella enterica serovars in the streptomycin-pretreated mouse model. Infect Immun 74: 632-644.

6. Stecher B, Robbiani R, Walker AW, Westendorf AM, Barthel M, et al. (2007) Salmonella enterica Serovar Typhimurium Exploits Inflammation to Compete with the Intestinal Microbiota. PLoS Biol 5: e244.

7. Stecher B, Hapfelmeier S, Muller C, Kremer M, Stallmach T, et al. (2004) Flagella and Chemotaxis Are Required for Efficient Induction of Salmonella enterica Serovar Typhimurium Colitis in Streptomycin-Pretreated Mice. Infect Immun 72: 4138-4150.

8. Hapfelmeier S, Muller AJ, Stecher B, Kaiser P, Barthel M, et al. (2008) Microbe sampling by mucosal dendritic cells is a discrete, MyD88-independent step in DeltainvG S. Typhimurium colitis. J Exp Med 205: 437-450.

9. Wang RF, Kushner SR (1991) Construction of versatile low-copy-number vectors for cloning, sequencing and gene expression in Escherichia coli. Gene 100: 195-199.

10. Mombaerts P, Mizoguchi E, Ljunggren HG, Iacomini J, Ishikawa H, et al. (1994) Peripheral lymphoid development and function in TCR mutant mice. Int Immunol 6: 1061-1070.

11. Chen J, Trounstine M, Alt FW, Young F, Kurahara C, et al. (1993) Immunoglobulin gene rearrangement in B cell deficient mice generated by targeted deletion of the JH locus. Int Immunol 5: 647-656.

12. Harriman GR, Bogue M, Rogers P, Finegold M, Pacheco S, et al. (1999) Targeted deletion of the IgA constant region in mice leads to IgA deficiency with alterations in expression of other Ig isotypes. J Immunol 162: 2521-2529.

13. Johansen FE, Pekna M, Norderhaug IN, Haneberg B, Hietala MA, et al. (1999) Absence of epithelial immunoglobulin A transport, with increased mucosal leakiness, in polymeric immunoglobulin receptor/secretory component-deficient mice. J Exp Med 190: 915-922.

14. Stecher B, Chaffron S, Kappeli R, Hapfelmeier S, Freedrich S, et al. (2010) Like will to like: abundances of closely related species can predict susceptibility to intestinal colonization by pathogenic and commensal bacteria. PLoS Pathog 6: e1000711.

15. Hughes JB, Hellmann JJ, Ricketts TH, Bohannan BJ (2001) Counting the uncountable: statistical approaches to estimating microbial diversity. Appl Environ Microbiol 67: 4399-4406.
